# Upregulation of CALD1 predicted a poor prognosis for platinum-treated ovarian cancer and revealed it as a potential therapeutic resistance target

**DOI:** 10.1186/s12864-024-10056-0

**Published:** 2024-02-16

**Authors:** Wei Li, Limei Huang, Nana Qi, Qinle Zhang, Zailong Qin

**Affiliations:** Genetic and Metabolic Central Laboratory, Birth Defect Prevention Research Institute, Maternal and Child Health Hospital, Children’s Hospital of Guangxi Zhuang Autonomous Region, Nanning, 530002 China

**Keywords:** CALD1, Ovarian cancer, Platinum resistance, Prognosis value, Target

## Abstract

**Background:**

Ovarian cancer (OC) has the worst prognosis among gynecological malignancies, most of which are found to be in advanced stage. Cell reduction surgery based on platinum-based chemotherapy is the current standard of treatment for OC, but patients are prone to relapse and develop drug resistance. The objective of this study was to identify a specific molecular target responsible for platinum chemotherapy resistance in OC.

**Results:**

We screened the protein-coding gene Caldesmon (CALD1), expressed in cisplatin-resistant OC cells in vitro. The prognostic value of CALD1 was evaluated using survival curve analysis in OC patients treated with platinum therapy. The diagnostic value of CALD1 was verified by drawing a Receiver Operating Characteristic (ROC) curve using clinical samples from OC patients. This study analyzed data from various databases including Gene Expression Omnibus (GEO), Human Protein Atlas (HPA), The Cancer Cell Line Encyclopedia (CCLE), The Cancer Genome Atlas (TCGA), GEPIA 2, UALCAN, Kaplan–Meier (KM) plotter, LinkedOmics database, and String. Different expression genes (DEGs) between cisplatin-sensitive and cisplatin-resistant cells were acquired respectively from 5 different datasets of GEO. CALD1 was selected as a common gene from 5 groups DEGs. Online data analysis of HPA and CCLE showed that CALD1 was highly expressed in both normal ovarian tissue and OC. In TCGA database, high expression of CALD1 was associated with disease stage and venous invasion in OC. Patients with high CALD1 expression levels had a worse prognosis under platinum drug intervention, according to Kaplan–Meier (KM) plotter analysis. Analysis of clinical sample data from GEO showed that CALD1 had superior diagnostic value in distinguishing patients with platinum "resistant" and platinum "sensitive" (AUC = 0.816), as well as patients with worse progression-free survival (AUC = 0.741), and those with primary and omental metastases (AUC = 0.811) in ovarian tumor. At last, CYR61 was identified as a potential predictive molecule that may play an important role alongside CALD1 in the development of platinum resistance in OC.

**Conclusions:**

CALD1, as a member of cytoskeletal protein, was associated with poor prognosis of platinum resistance in OC, and could be used as a target protein for mechanism study of platinum resistance in OC.

**Supplementary Information:**

The online version contains supplementary material available at 10.1186/s12864-024-10056-0.

## Background

The ovarian cancer (OC) is the most lethal among gynecologic malignancies, exhibiting the poorest prognosis compared to other gynecologic malignancies [1, 2]. Due to the lack of reliable screening methods in the early stages of the disease, the clinical presented symptoms is unclear, resulting in a majority of OC cases being diagnosed at advanced stages (FIGO stage III-IV). The five-year overall survival rate for patients with advanced-stage disease is approximately 40%–45% [3]. The current standard of treatment for OC is cell reduction surgery based on platinum-based chemotherapy. First line chemotherapy includes 6 cycles of platinum-based regimen [4]. The most active therapeutic agents against newly diagnosed OC were platinum analogues (either cisplatin or carboplatin) with the addition of a taxane (either paclitaxel or docetaxel). Approximately 20–30% of patients with OC exhibit intrinsic resistance to platinum-based chemotherapy within 6 months after their standard treatment, although a high rate of remission is achieved following first-line therapy [5]. Patients classified as "platinum resistant" experience relapse within 6 months after completing first-line therapy, which subsequently leads to shortened progression-free survival and low response rates (< 15%) to subsequent chemotherapy [6]. Chemotherapy resistance ultimately emerges as a fatal determinant in OC, necessitating the identification of novel genes to serve as prognostic markers for primary platinum-based chemoresistance in OC.

Cisplatin and carboplatin chemotherapy widely used in current practice were shown to shrink the cancer in 10% to 30% of patients. A number of studies have shown that in advanced cancers, such as pancreatic and gastric cancers, overall survival (OS) after chemotherapy is significantly increased relative to best supportive care (BSC). These forms of chemotherapy include gemcitabine and platinum-based combination, 5-fluorouracil and platinum combination, taxane-platinum combinations [7, 8]. In addition, platinum-based chemotherapy using carboplatin in the adjuvant or neoadjuvant setting improves long-term outcomes of disease-free survival (DFS) and overall survival (OS) in early triple-negative breast cancer [9]. It can be seen that platinum based therapies are very important in the treatment of cancers. Cisplatin chemotherapy when combined with other drugs has been shown to prolong survival by a few months compared with cisplatin alone, but with the cost of increased side effects [10]. The development of resistance to therapy in OC is a significant hindrance to therapeutic efficacy. Despite using chemotherapy, the 5 year survival rate of patients with OC remains less than 50%, mainly due to chemotherapy resistance. Meanwhile, the numerous side effects associated with platinum-based therapies, such as neutropenia, anaemia and thrombocytopenia, the need for novel biomarkers and methods in the treatment of platinum-resistant OC has become increasingly urgent. Some future strategies to improved therapeutic responses for OC treatment include effective targeted therapy, usage of PARP inhibitors, combination therapy, immunotherapy, and usage of chemo-sensitizers [11]. There are some genes working against apoptosis in cancer, which allows cancerous cells to flourish instead of being killed off. Bcl-2 has anti-apoptotic effect and is highly expressed in OC. Clinical trials have shown that the treatment of OC with an inhibitor of Bcl-2 improves the response to cisplatin. Immunotherapy involves various methods enhancing immune system. The principle is that anti-tumor lymphocytes from healthy adults and patients are used in treatment using adoptive cell transfer to stimulate cancer decline. But there is not yet any FDA approval for immune therapies for OC because the success rate for immunotherapy of OC treatment is very low. Besides, cancer vaccine therapeutic investigation is an actively growing area in OC researches. Vaccines used in cancer therapy will activate the immune cells for the elimination of cancerous cells. In terms of inhibitor, olaparib is a PARP inhibitor that only works on cells where the BRCA pathway is blocked. The limitation is that olaparib is only used for cancer patients with BRCA gene mutations but only a small percentage of patients with ovarian cancer have mutations in the BRCA gene. Numerous new strategies are being studied to try to overcome this chemical resistance, including combining platinum based chemotherapy with new molecularly targeted drugs, such as bevacizumab or olaparib [12]. Searching for new biomarkers to improve survival rates and ameliorate the quality of life in platinum-resistant OC patients is still the goal of follow-up researches.

Cytoskeletal proteins are closely implicated in tumorigenesis and migration, with numerous studies highlighting the pivotal roles of many cytoskeletal proteins, particularly those candidate genes associated with actin during tumor progression [13–15]. Caldemon (CALD1) is a cytoskeletal protein primarily located in the fine filaments of smooth muscle, linking actin, calmodulin, tropomyosin and myosin. The single CALD1 gene encodes high molecular weight CaD (h-CaD) and low molecular weight CaD (l-CaD) through variable splicing. Studies have demonstrated that CALD1 plays a crucial role in regulating the actomyosin systolic system, affecting various cellular functions such as cell movement, invasion, migration and proliferation [16, 17]. The expression of CALD1 has been linked to the development of several benign and malignant tumors including uterine leiomyoma [18], bladder cancer [19, 20], colon cancer [21], prostate cancer [22]. Despite its confirmed expression in various solid tumors, few studies have investigated the expression profile or function of CALD1 in OC so far. Furthermore, no studies have reported on the effect of CALD1 on platinum chemotherapy sensitivity for OC. In this study, we aim to investigate both the expression and function of cytoskeletal protein CALD1 with regards to platinum-based chemoresistance in OC.

## Results

### Significantly up-regulated and down-regulated different expression genes (DEGs) were identified across five datasets

**Fig. 1:**
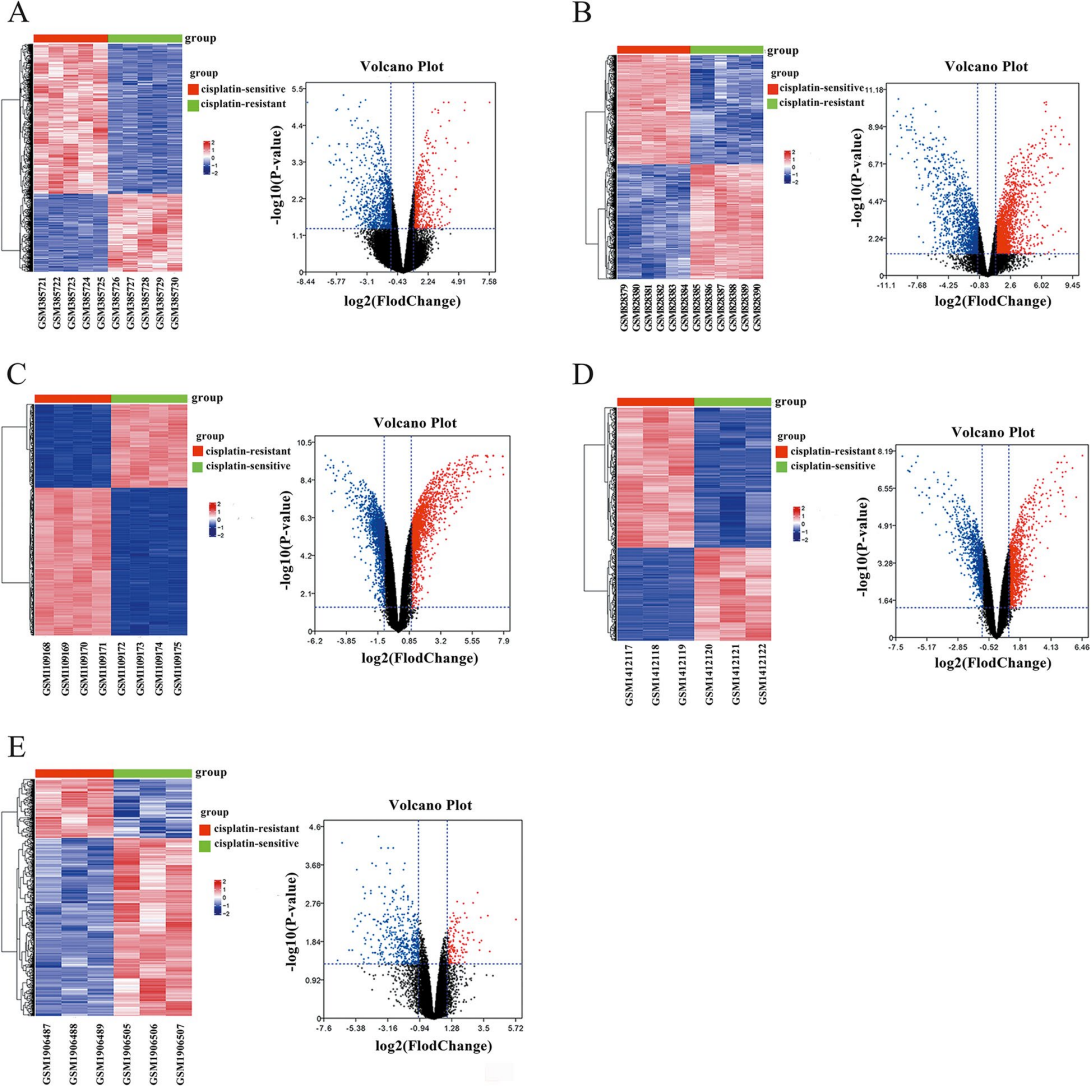
Identification of DEGs in GSE 15372, GSE33482, GSE45553, GSE58470 and GSE73935. Heat maps and volcano plots of DEGs obtained from the GSE15372 dataset (**A**), GSE33482 dataset (**B**), GSE45553 dataset (**C**), GSE58470 dataset (**D**), GSE73935 dataset (**E**). Hierarchical clustering analysis of DEGs, which were differentially expressed between cisplatin-sensitive and cisplatin-resistant cell lines of OC (> twofold different in expression and *P* < 0.05). Volcano plots were constructed using fold-change value and adjusted P. The red point in the plot represents the up- regulated genes and green point represents the down- regulated genes with statistical significance

Across five datasets published in GEO, we screened DEGs between cisplatin-sensitive and cisplatin-resistant cell lines in vitro. Hierarchical clustering analysis and volcano plots were used to assess variation in gene expression. The heat maps of GSE 15372, GSE33482, GSE45553, GSE58470 and GSE73935 are shown in Fig. 1 respectively. Volcano plots were also generated to better visualize the differentially expressed genes (Fig. 1). In GSE 15372, a total of 528 genes were significantly up-regulated while 1166 genes were down-regulated. Similarly, in GSE 33482, a large number of genes exhibited significant differential expression in OC cell lines with 2060 up-regulated and 1546 down-regulated genes identified. Using the same approach, we identified 1822 significantly up-regulated and 1865 significantly down-regulated genes in GSE45553 as well as 1182 up-regulated and 1060 down-regulated genes in GSE58470. Finally, through the same method applied to GSE73935 data set, we found that there were 140 up-regulated and 516 down-regulated genes. The conditions for hierarchical clustering analysis were > twofold different in expression and *P* < 0.05. The heat map shows the top 1000 genes that are statistically significant and more than 2 × differentially expressed. Volcano plots were constructed using fold-change value and adjusted P. Heatmaps was plotted using the OmicShare tools, a free online platform for data analysis (www.omicshare.com/tools).

### Screening target gene with venn plot

**Fig. 2:**
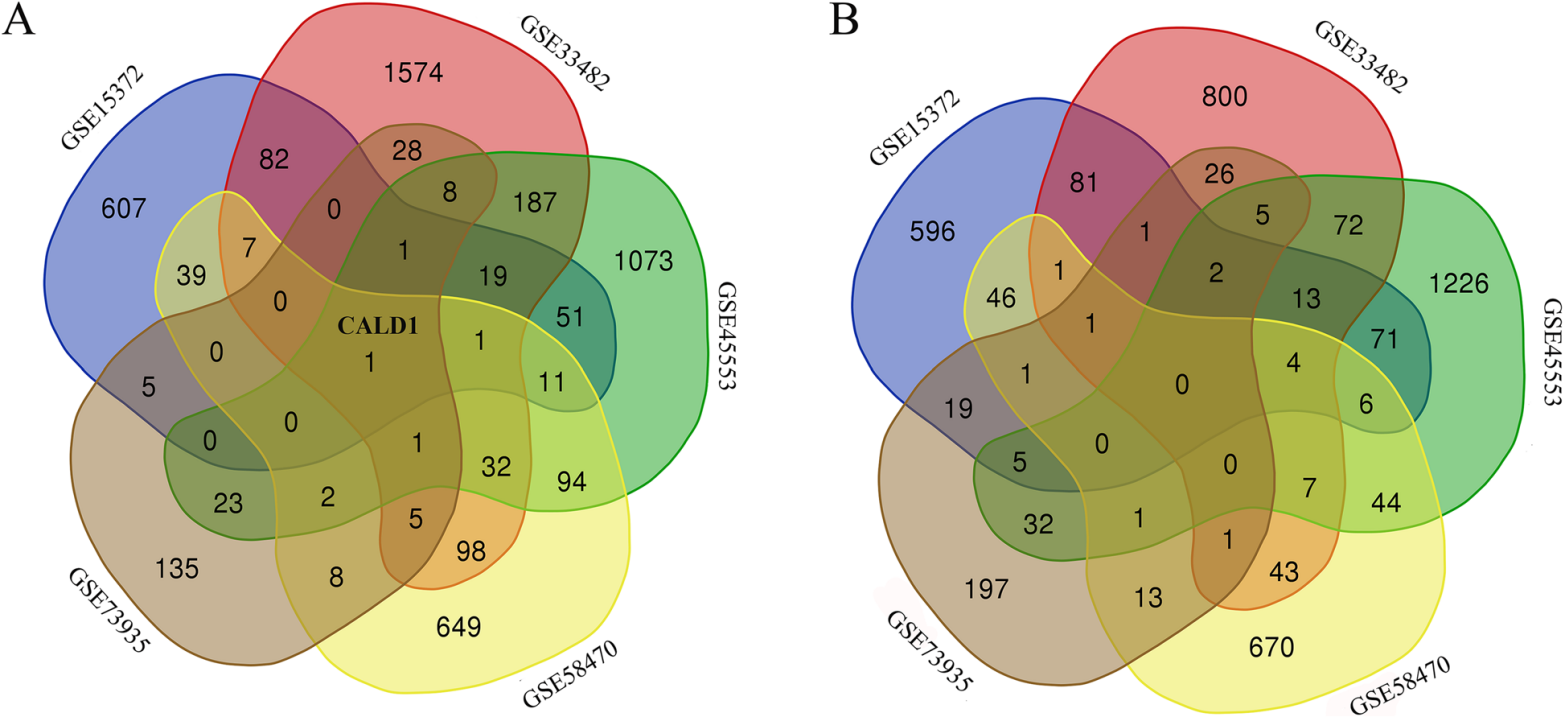
Screening common expressed gene in DEGs from 5 datasets. CALD1 was the only common gene among the 5 groups of up-regulated DEGs (**A**), and no common genes were found in the down-regulated DEGs (**B**)

By employing Venn diagrams as a tool, we identified CALD1 as the commonly up-regulated gene in platinum-resistant cells across all 5 datasets, when compared to sensitive or parent cells (Fig. 2A). There was no common gene that was down-regulated across all 5 datasets. CALD1 is the only DEG used for follow-up studies (Fig. 2B).

### Expression profile of CALD1 across various organizations and cancers

**Fig. 3:**
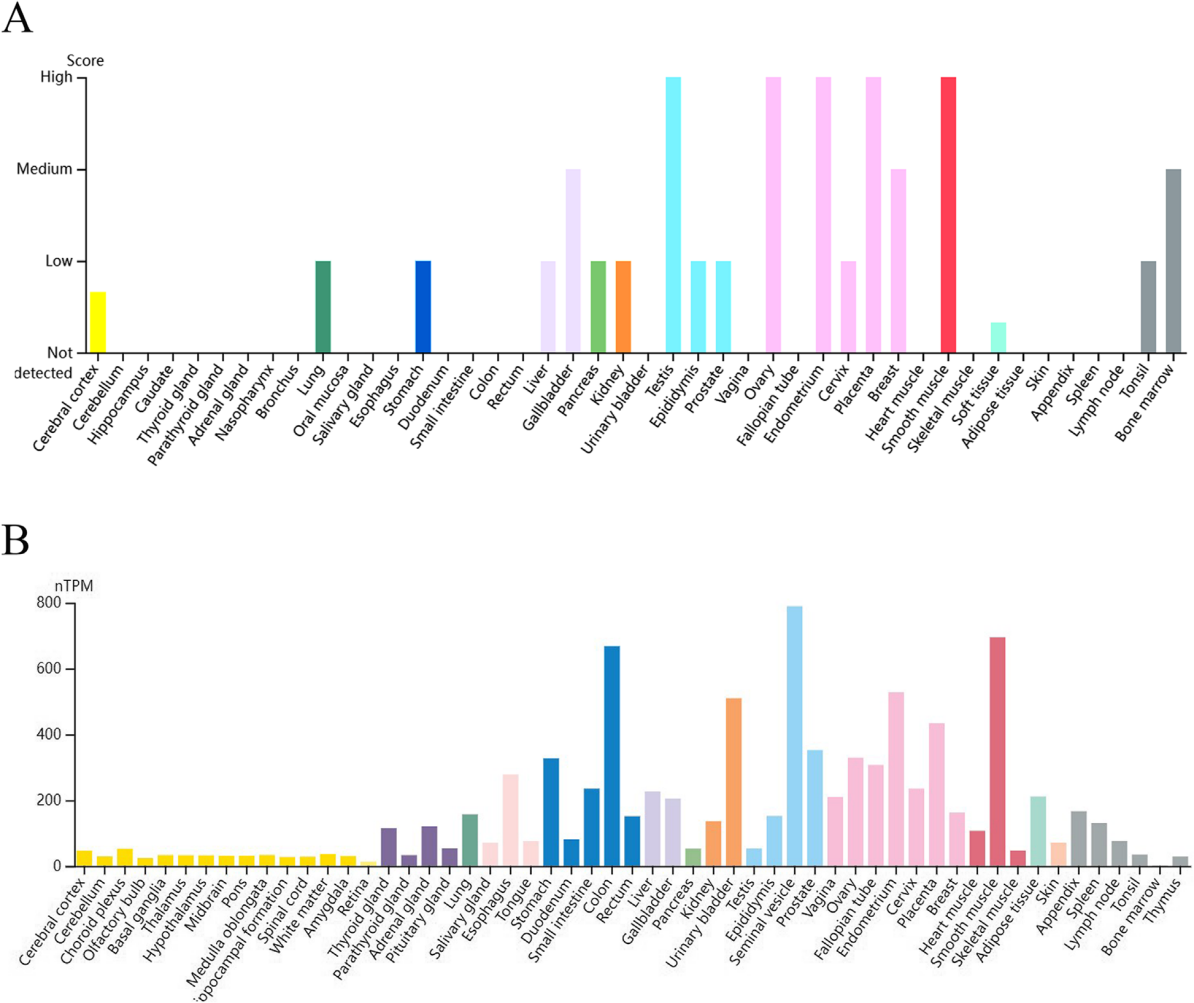
Expression data of CALD1 derived from ovarian cell lines recorded in the HPA databases were utilized. Protein expression data (**A**) and RNA-seq data (**B**) of CALD1 across various tissues were examined. Ovarian protein expression data of CALD1 (score: High) were obtained from ovarian stroma cells. RNA expression data of CALD1 based on the consensus dataset (nTPM:339.1) were obtained from stromal cells, smooth muscle cells and other cell types of ovary

**Fig. 4:**
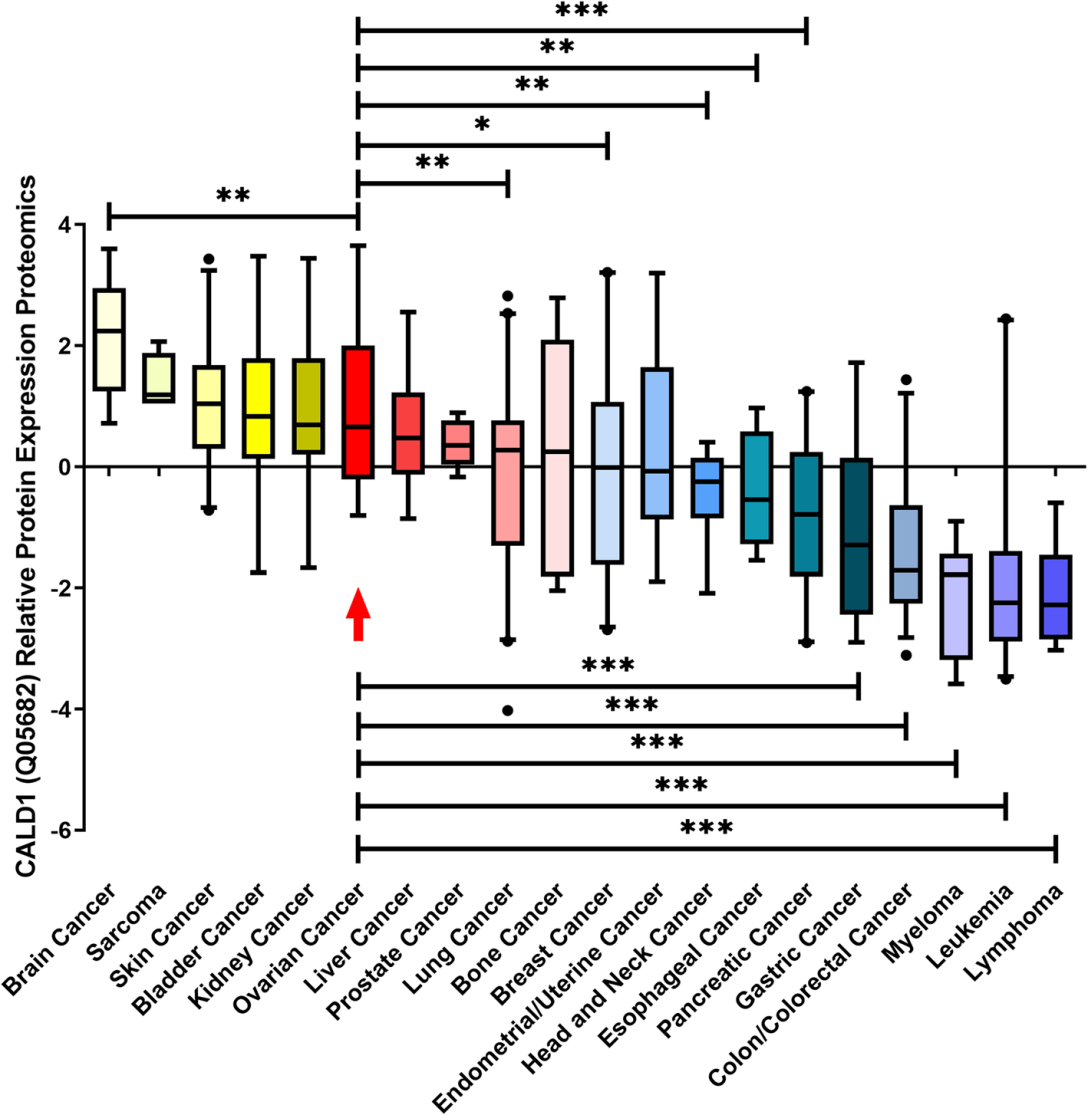
The CALD1 protein expression level of various kinds of cancers analyzed by CCLE. The expression of CALD1 in ovarian cancer was higher than that of many cancer types like lung cancer, breast cancer, head and neck cancer, esophageal cancer, pancreatic cancer, gastric cancer, colon/colorectal cancer, myeloma, leukemia, lymphoma. The results were statistically significant. The red box with the red arrow represented ovarian tumor.(* indicates *P* < 0.05, ** indicates *P* < 0.01, *** indicates *P* < 0.001)

**Fig. 5:**
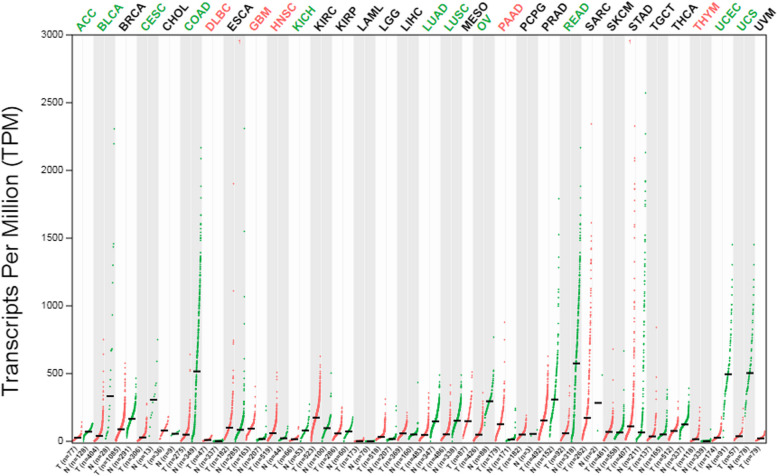
Pan cancer analysis of CALD1 between tumor vs non-tumor tissues was performed by GEPIA 2. The gene expression profile across all tumor samples and paired normal tissues was presented using dot plot. Each dots represent expression of samples. (Ret dots represent tumor samples, green dots represent paired normal tissues. T: tumor, N: normal samples; |Log2FC| Cutoff: 1, q-value Cutoff: 0.01)

**Fig. 6:**
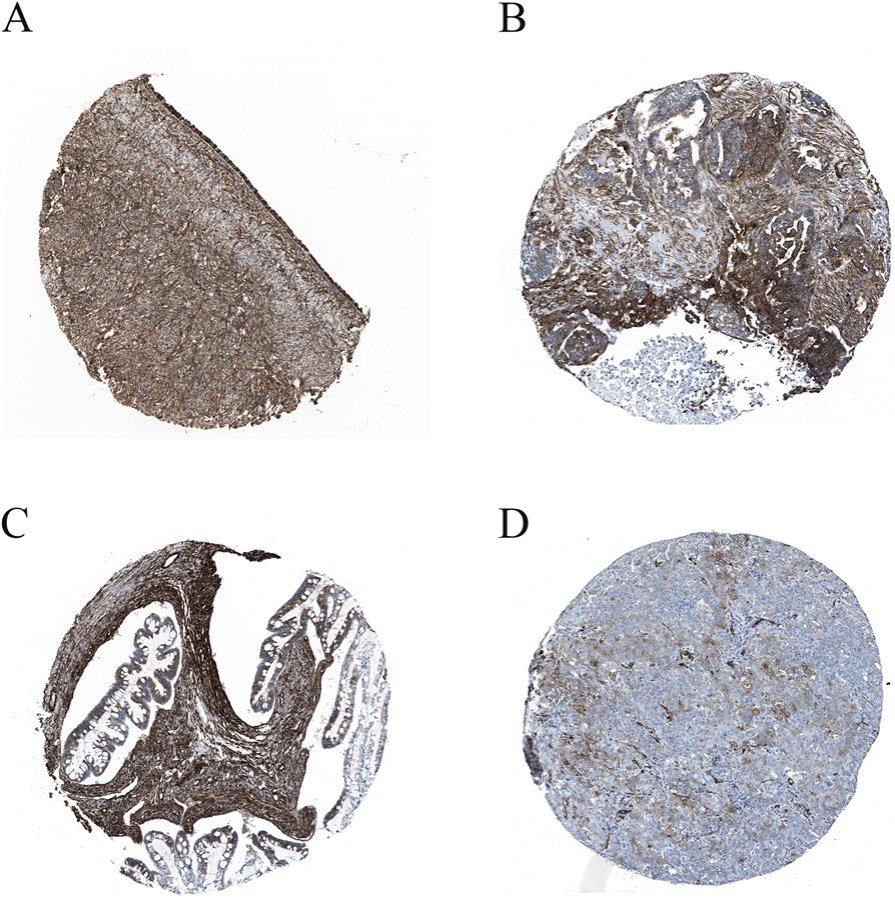
The Immunohistochemistry (IHC) of CALD1 in different kinds of OC and corresponding normal tissues based on the Human Protein Atlas (HPA). **A** Normal ovarian tissue (Staining: High; Intensity: Strong; Quantity: > 75%). **B** Ovarian serous cystadenocarcinoma (Staining: High; Intensity: Strong; Quantity: > 75%). **C** Ovarian mucinous cystadenocarcinoma (Staining: High; Intensity: Strong; Quantity: > 75%). **D** Endometroid carcinoma of ovary (Staining: Medium; Intensity: Moderate; Quantity: 75%-25%)

To assess the expression profile of CALD1, we analyzed RNA-seq and protein expression data from ovary cell lines available in the HPA databases. The HPA database revealed that CALD1 was expressed in multiple tissues with higher levels observed in the ovary compared to other tissues (Fig. 3A). The RNA expression levels were not as high as protein levels, yet higher than those observed in several other tissues including cervix, thyroid, vagina, fallopian tube, breast, heart muscle, liver, skin, etc. (Fig. 3B). The proteomic analysis of CALD1 protein expression in the CCLE revealed that ovarian tumor exhibited higher levels of CALD1 expression compared to various primary tumors, including lung cancer, breast cancer, head and neck cancer, esophageal cancer, pancreatic cancer, gastric cancer, colon/colorectal cancer, myeloma, leukemia, lymphoma, and the data were statistically significant (Fig. 4). Pan cancer analysis of CALD1 was performed by GEPIA 2 showed in Fig. 5. Compared to matched normal samples, the expression of CALD1 was up-regulated in glioblastoma multiforme, head and neck squamous cell carcinoma, kidney renal clear cell carcinoma, pancreatic adenocarcinoma, etc., and was down-regulated in bladder urothelial carcinoma, breast invasive carcinoma, cervical squamous cell carcinoma and endocervical adenocarcinoma, colon adenocarcinoma, lung adenocarcinoma, lung squamous cell carcinoma, ovarian serous cystadenocarcinoma, prostate adenocarcinoma, uterine corpus endometrial carcinoma, uterine carcinosarcoma, etc. Subsequently, we searched the immunohistochemistry (IHC) of various types of OC in the HPA database. Here, the results of antibody HPA017330 staining revealed that CALD1 was intensively expressed in ovary normal tissue (ovarian stroma cells) (Fig. 6A), and was intensively or moderately expressed in ovarian serous cystadenocarcinoma, ovarian mucinous cystadenocarcinoma, and endometroid carcinoma of ovary (Fig. 6BCD). The brief informations of these patients was listed in the following Table 1.

**Table 1:** IHC informations of patients of various OC

Variable	Age		Classification			Staining				Intensity			
	< 70	≥ 70	M-84413^a^	M-83803^b^	M-84703^c^	High	Medium	Low	/^d^	High	Medium	Low	/^d^
number	9	3	6	2	4	2	7	2	1	2	8	1	1

^a^M-84413: Cystadenocarcinoma, serous, NOS

^b^M-83803: Carcinoma, endometroid

^c^M-84703: Cystadenocarcinoma, mucinous, NOS

^d^Not detected

### Analysis of association between CALD1 and OC disease stages

**Fig. 7:**
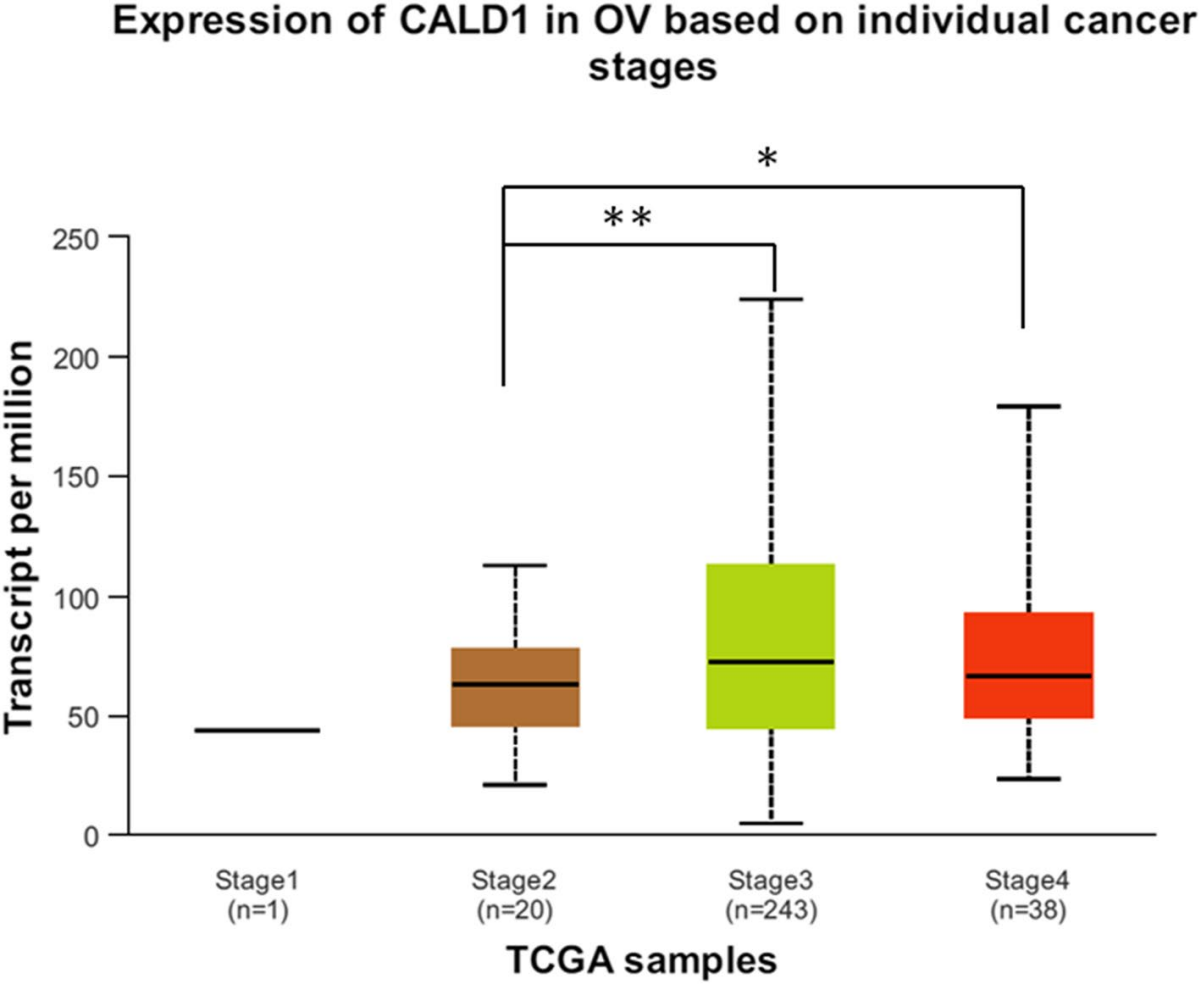
Expression of CALD1 in ovarian serous cystadenocarcinoma based on individual cancer stages. Stage 1 exhibited the smallest sample size, limited to one case, potentially indicating that early detection of ovarian cancer is more challenging. The expression level of CALD1 at stage3 and stage 4 was significantly increased compared with stage2 (*P* < 0.05), while stage 4 had no statistical significance compared with stage 3. This result suggests CALD1 is associated with the disease stage. ( * indicates *P* < 0.05, ** indicates *P* < 0.01)

Using cohorts of TCGA, CALD1 expression profile based on individual OC stages was obtained from UALCAN (Fig. 7). A total of 302 samples participated in the analysis, including stage 1 (n = 1), stage 2 (n = 20), stage 3 (n = 243) and stage 4 (n = 38). With the progression of the disease, the expression of CALD1 increased. Compared with stage 2, CALD1 demonstrated a significant up-regulation in stage 3 (*P* = 0.0086) and 4 (*P* = 0.024) respectively, with statistical significance. Between stage 3 and stage 4, the expression of this protein was not statistically significant. With the escalation of malignant degree of OC, the expression of CALD1 is up-regulated, which further proves that the elevated expression of this molecule is associated with the disease stage and the occurrence and development of OC, potentially facilitating its advancement.

### Relationships between CALD1 and clinicopathological characteristics of patients with OC

In this study, the clinical and molecular characteristics of all patients from the TCGA database are summarized in Table 2. No significant differences in age, grade, longest dimension, anatomic neoplasm subdivision, residual tumor, and lymphatic invasion (*p* > 0.05) were found among the groups of each characteristics. Significant differences were determined in the clinical stage and venous invasion. In stageIII-IV of OC, the expression of CALD1 was significantly increased compared with stageI-II (*P* = 0.034). This result was consistent with that obtained by the UALCAN online analysis tool. Notably, expression of CALD1 in the group with venous invasion was significantly elevated compared to the group lacking venous invasion (*P* < 0.001). This indicates that CALD1 is likely to be involved in cancer cell invasion and metastasis.

**Table 2:** Correlations between expression of CALD1 and clinicopathologic characteristics in OC from TCGA cohort (n = 379)

Clinical features	Case	CALD1 level (mean ± SD)	*p* value
**Age at diagnosis (years)**			
≤ 60	208	4.2896 ± 0.8494	0.474
> 60	171	4.2229 ± 0.9611	
**Clinical stage**			
I-II	24	3.8833 ± 0.6890	0.034^*^
III-IV	352	4.2831 ± 0.9046	
Unknown	3		
**Grade**			
G1-G2	46	4.2779 ± 0.9729	0.888
G3-G4	323	4.2577 ± 0.8960	
Unknown	10		
**Longest dimension**			
≤ 1	84	4.2222 ± 0.9496	0.445
1–2	188	4.2975 ± 0.9387	
≥ 2	43	4.4390 ± 0.6436	
Unknown	64		
**Anatomic neoplasm subdivision**			
Bilateral	255	4.2740 ± 0.9351	0.446
Unilaterral	102	4.1931 ± 0.8228	
Unknown	22		
**Residual tumor**			
0	67	4.1610 ± 0.7174	0.706
1–10 mm	171	4.3079 ± 0.9005	
11–20 mm	27	4.2219 ± 0.6972	
> 20 mm	70	4.2757 ± 1.0448	
Unknown	44		
**Lymphatic invasion**			
Yes	101	4.2238 ± 0.9285	0.736
No	48	4.1707 ± 0.8225	
Unknown	230		
**Venous invasion**			
Yes	32	4.2038 ± 0.6325	< 0.001^***^
No	35	3.5051 ± 0.4561	
Unknown	312		

*SD* Standard Deviation

*Note*: ^*^ *P* < 0.05, ^***^ *P* < 0.001

### High expression of CALD1 is associated with poor prognosis with Kaplan–Meier (KM) plotter analysis

**Fig. 8:**
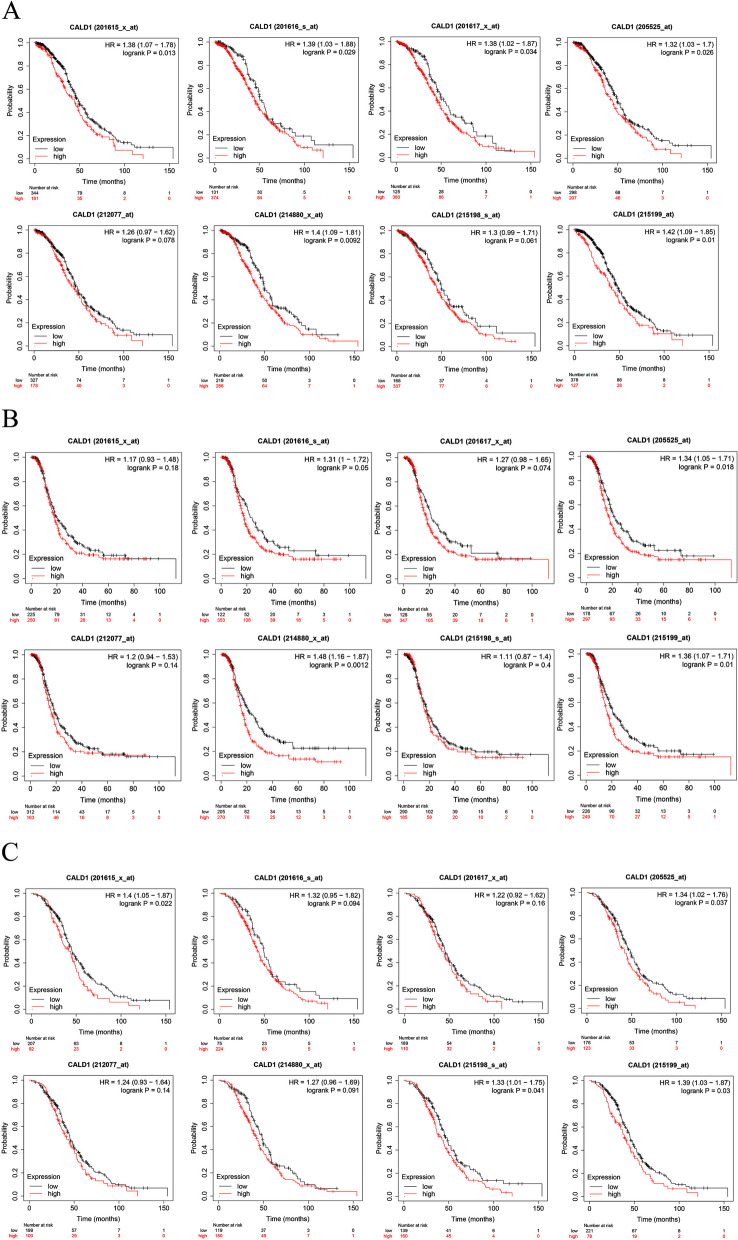
The correlation between CALD1 expression and OS (A), PFS(B), PPS(C) in serous OC patients treated with platin-based chemotherapy from TCGA, which were displayed by 8 Affy ID. The red curve line represents the high expression of CALD1 and the black curve represents the low expression of CALD1. Each Affy ID corresponds to a graph with HR value and P value. *P* < 0.05 indicates statistical significance

**Fig. 9:**
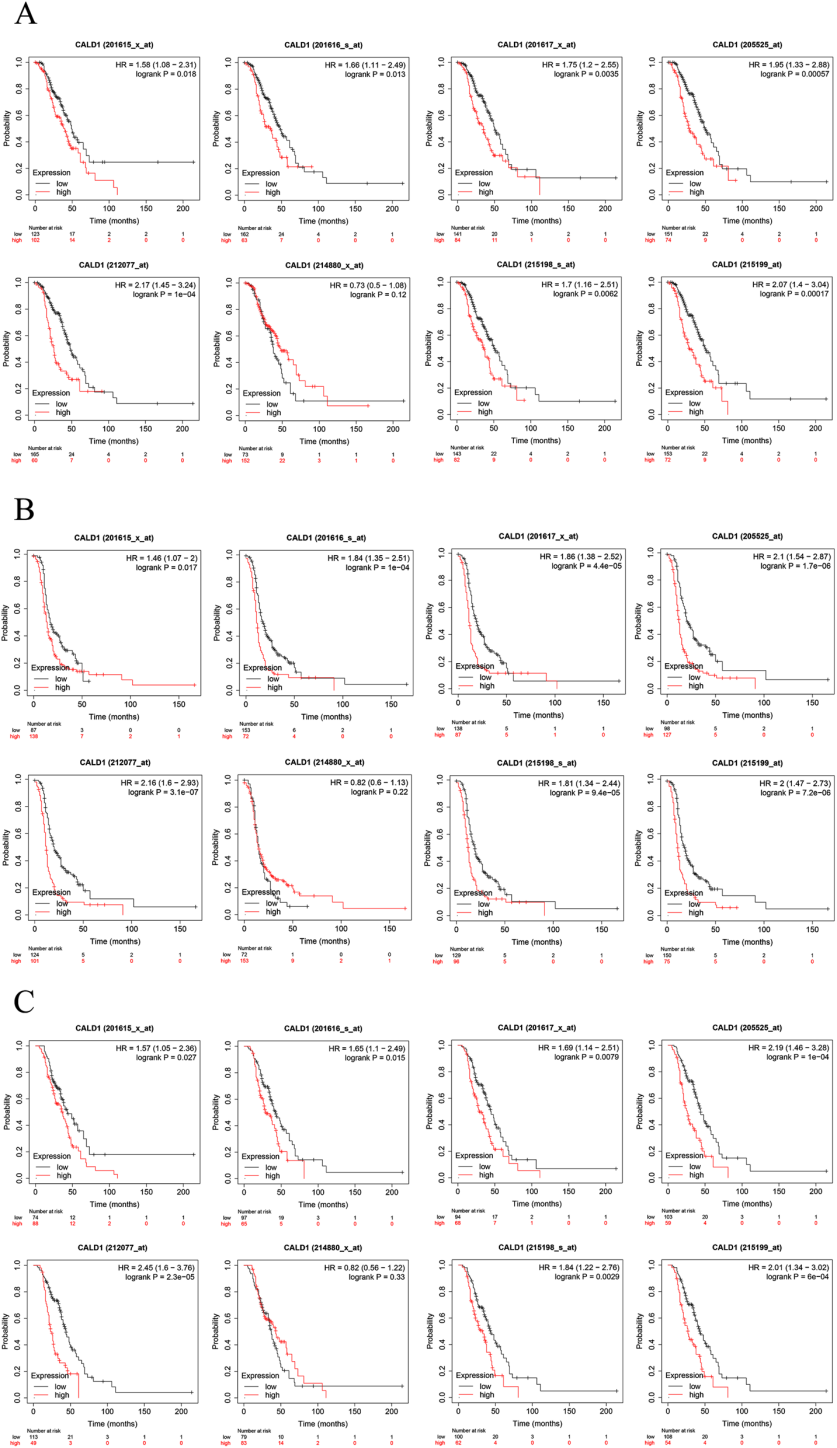
The correlation between CALD1 expression and OS (**A**), PFS(**B**), PPS (**C**) in serous OC patients treated with platin-based chemotherapy from GSE9891, which were displayed by 8 Affy ID. The red curve line represents the high expression of CALD1 and the black curve represents the low expression of CALD1. Each Affy ID corresponds to a graph with HR value and P value. *P* < 0.05 indicates statistical significance

**Fig. 10:**
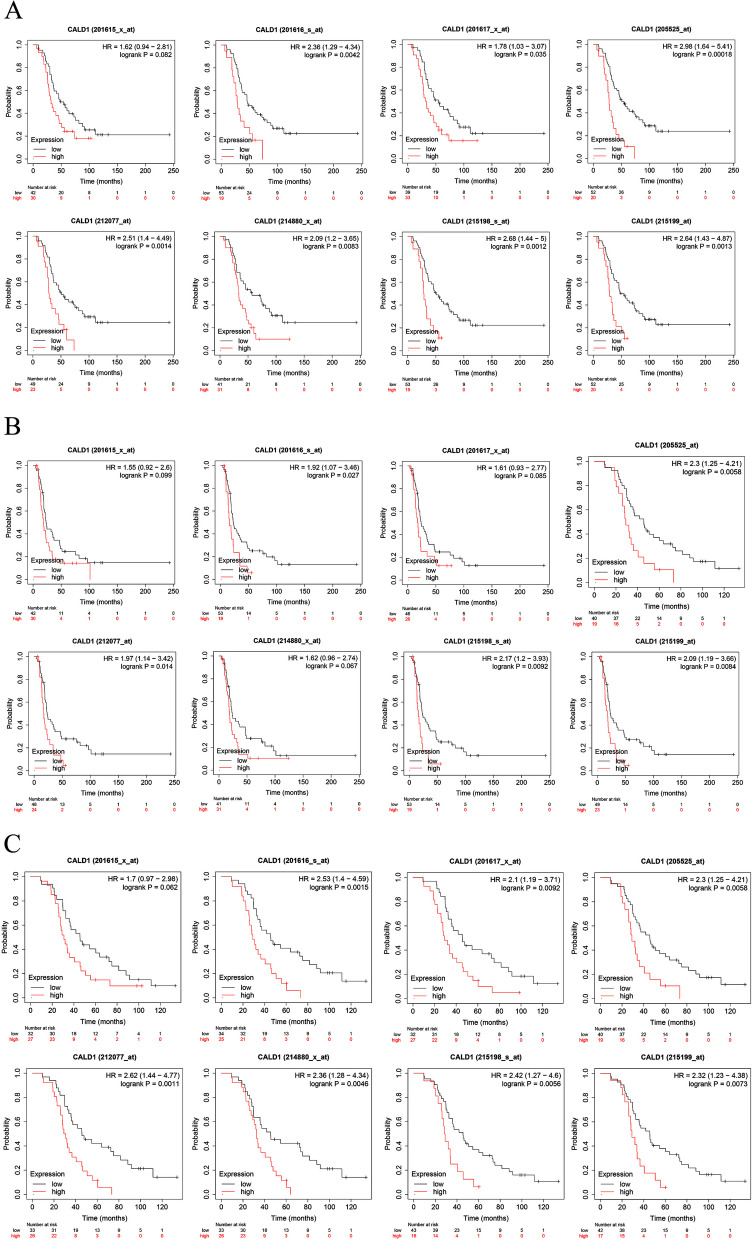
The correlation between CALD1 expression and OS (**A**), PFS (**B**), PPS (**C**) in serous OC patients treated with platin-based chemotherapy from GSE 26193, which were displayed by 8 Affy ID. The red curve line represents the high expression of CALD1 and the black curve represents the low expression of CALD1. Each Affy ID corresponds to a graph with HR value and P value. *P* < 0.05 indicates statistical significance

The prognostic value of CALD1 in serous OC was assessed using Kaplan–Meier plotter for patients who received platinum-based chemotherapy. To comprehensively investigate the correlation between CALD1 expression and overall survival (OS), progression-free survival (PFS), and post-progression survival (PPS), “all probe sets per gene” was utilized to generate Kaplan–Meier plots. In most cases, CALD1 expression was significantly correlated with poor OS, PFS and PPS across different Affy ID (201615_x_at, 201616_s_at, 201617_x_at, 205525_at, 212077_at, 214880_x_at, 215198_s_at, 215199_at) in TCGA (Fig. 8), GSE9891(Fig. 9) and GSE 26193 (Fig. 10) (*P* < 0.05). The screening results suggest that the higher expression of CALD1 in OC patients using platin-based chemotherapy is most likely significantly correlated with the worse OS, PFS and PPS (*P* < 0.05). These results suggest that CALD1 may play a crucial role in promoting the development of OC and is significantly associated with poor prognosis of cancer.

### Functional enrichment analysis of the co-expression genes associated with CALD1

**Fig. 11:**
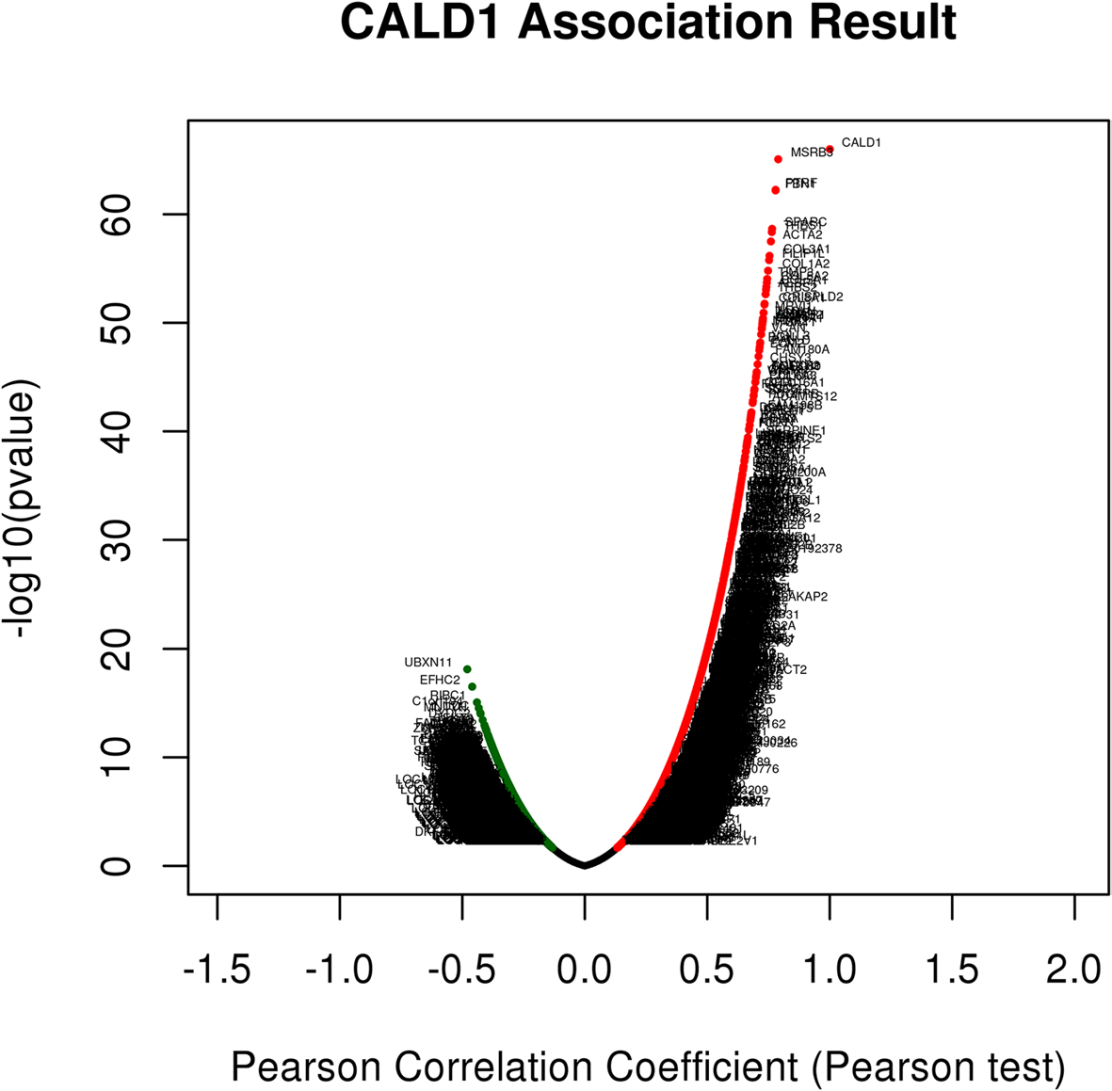
Co-expressed genes in conjunction with CALD1 of ovarian serous cystadenocarcinoma were analyzed in LinkedOmics. Genes positively correlated with CALD1 are represented by red dots, while negatively correlated genes are represented by green dots. Based on Pearson test, 4084 co-expression genes displayed positively signifcant correlations with CALD1, and 4295 genes were negatively signifcant correlations with CALD1. (FDR ≤ 0.05, *P* ≤ 0.05)

The co-expressed genes in conjunction with CALD1 were analyzed by LinkedOmics database (see Additional file 1). As shown in Fig. 11, a total of 9065 genes represented by red dots were positively correlated with CALD1, while 10,966 genes represented by green dots were negatively correlated with CALD1 in ovarian serous cystadenocarcinoma. Among the genes correlated with CALD1 expression, 4084 co-expression genes displayed positively signifcant correlations with CALD1, and 4295 genes were negatively signifcant correlations with CALD1. (FDR ≤ 0.05, *p* ≤ 0.05).

**Fig. 12:**
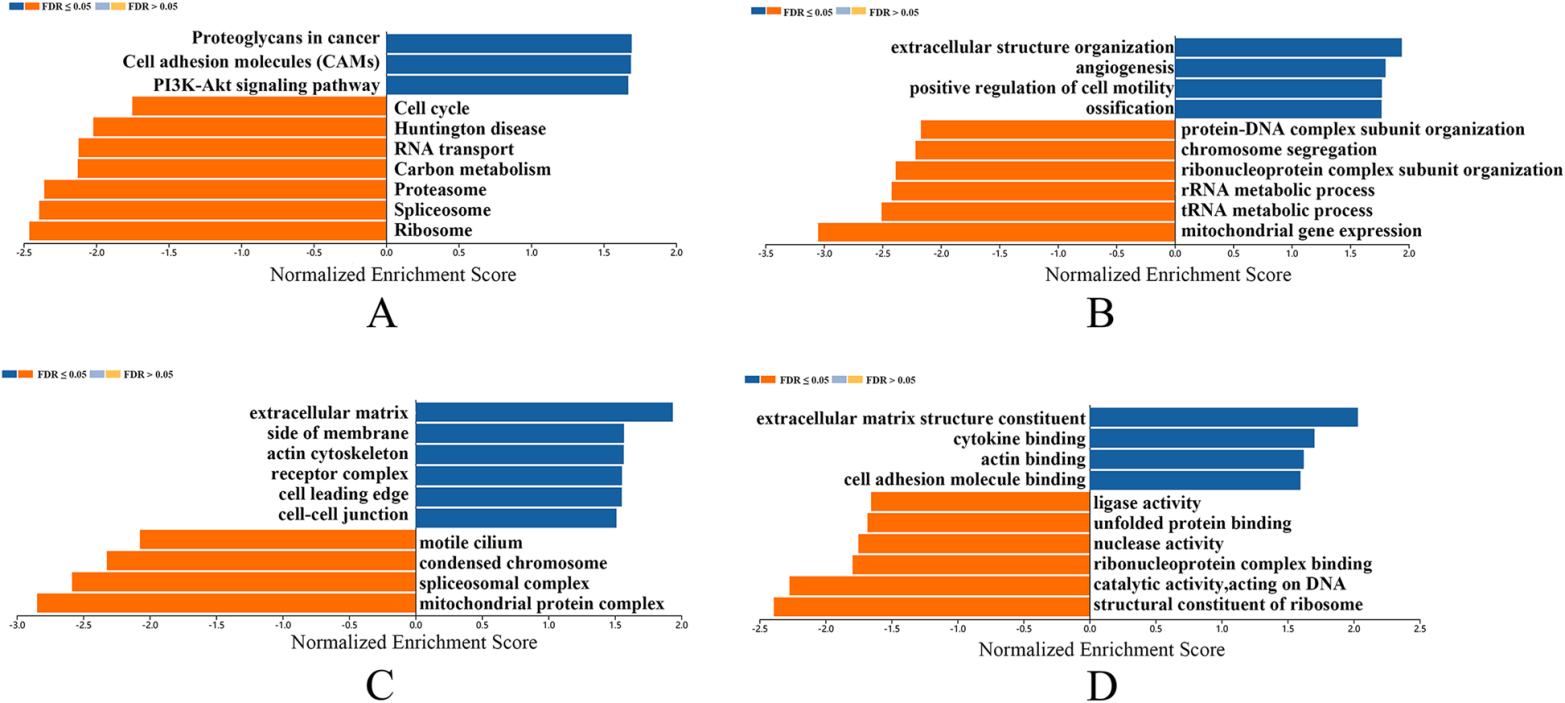
Function enrichment analysis of CALD1 in OC (LinkedOmics). **A** KEGG pathways analysis. **B** Biological process analysis. **C** Cellular component analysis. **D** Molecular function analysis. Both orange and dark blue bands represent FDR ≤ 0.05. In this analysis, both P and FDR values were less than 0.05with statistical significance

Subsequently, to further investigate the potential molecular mechanism and biological function of CALD1 in ovarian serous cystadenocarcinoma, the LinkInterpreter module of LinkedOmics were utilized to analyse function enrichment. Using Gene Set Enrichment Analysis (GSEA), the KEGG pathway indicated that CALD1 and its related genes were primarily associated with Proteoglycans in cancer, Cell adhesion molecules (CAMS), PI3K-Akt signaling pathway, Ribosome, Spliceosome, Proteasome, etc. (Fig. 12A). GO_BP (biological process) was mainly related to extracellular structure organization, angiogenesis, positive regulation of cell motility, mitochondrial gene expression, tRNA metabolic process, rRNA metabolic process, etc. (Fig. 12B). GO_CC (cell component) was mainly expressed in extracellular matrix, side of membrane, mitochondrial protein complex, spliceosomal complex, etc. (Fig. 12C). GO_MF (molecular function) was mainly associated with extracellular matrix structural constituent, cytokine binding, actin binding, structural constituent of ribosome, catalytic activity, acting on DNA, etc. (Fig. 12D). The analysis above had statistical significance (*P* < 0.05).

### PPI Network for CALD1 with strong positive correlation proteins

In this study, we focused on the identification of co-expressed genes that exhibited a strong positive correlation with CALD1. Using a Pearson correlation coefficient threshold of 0.6, we identified 140 proteins that were significantly and positively associated with CALD1 (FDR ≤ 0.05, *p* ≤ 0.05) (see Additional file 2).

**Fig. 13:**
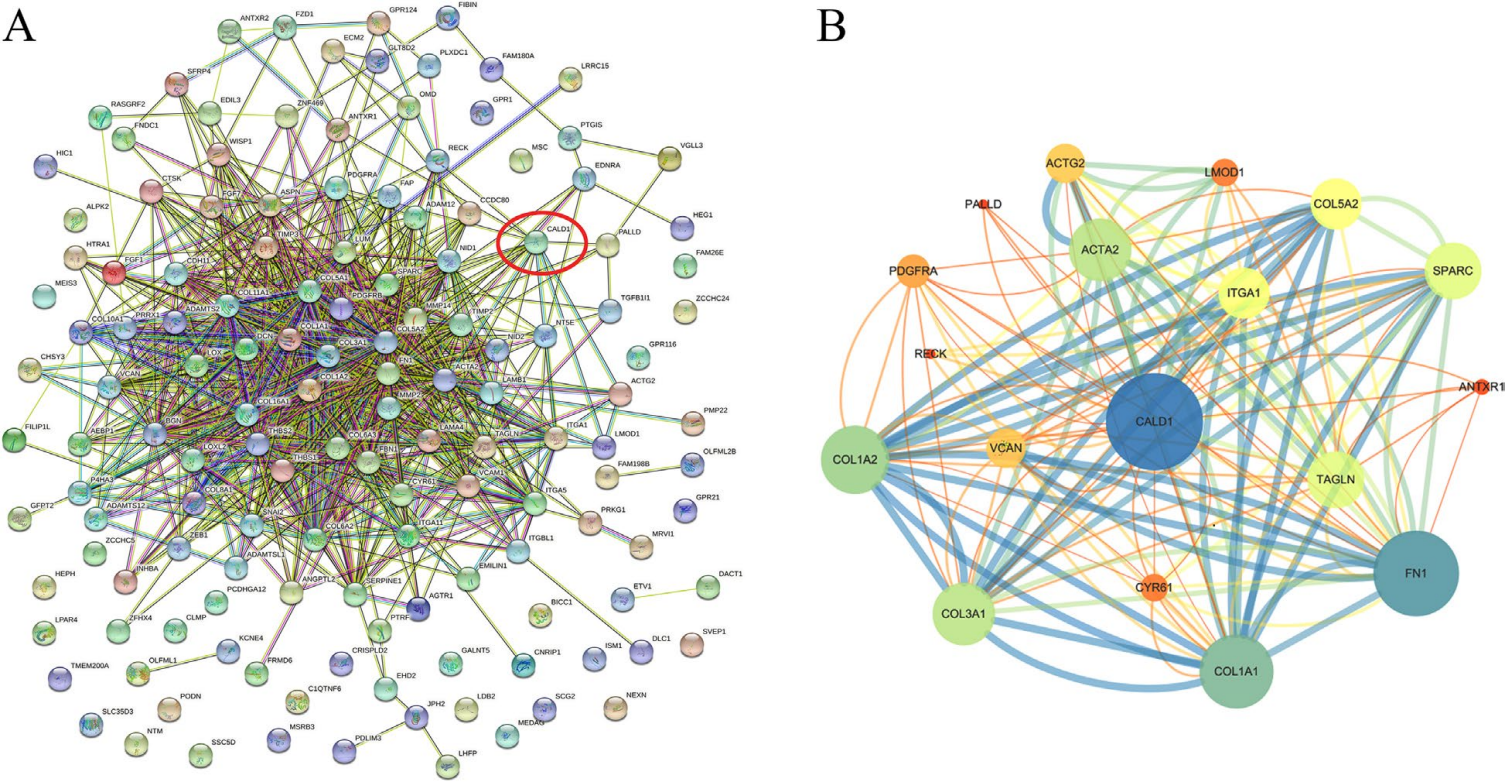
PPI network diagram was generated using String Database (https://string-db.org). **A** PPI network map of 141 DEGs (*P* < 0.05). The line thickness indicated the strength of data support, including experiments, text-mining, databases, co-expression, neighborhood, gene fusion, and co-occurrence. The protein shown in the red circle is CALD1. **B** The CALD1 related network extracted from (A) using the MCODE plugin in Cytoscape (version: 3.7.1). The wider the edge and circle size, the stronger the co-expression is; the darker the color, the lower combined-score is

The 141 genes were imported into the PPI network, resulting in the identification of 141 nodes and 854 edges (Fig. 13A) (PPI enrichment *p*-value: < 1.0e-16). The thicker connection line indicates higher reliability. Besides, we utilized software Cytoscape 3.7.1 to obtain visualization of proteins interactions network which interact directly with CALD1 (Fig. 13B). The related molecules that directly interact with CALD1 include ACTA2 (actin alpha 2), LMOD1 (leiomodin-1), PALLD (palladin), COL5A2 (collagen type V, alpha 2), ACTG2 (actin gamma 2), PDGFRA (platelet derived growth factor receptor alpha), ITGA1 (integrin, alpha 1), SPARC (secreted protein acidic and cysteine rich), RECK (reversion-inducing-cysteine-rich protein with Kazal motifs), COL1A2 (collagen, type I, alpha 2), VCAN (versican), ANTXR1 (anthrax toxin receptor 1), TAGLN (transgelin), CYR61 (cysteine-rich, angiogenic inducer, 61), COL3A1 (collagen, type III, alpha 1), FN1 (fibronectin1), and COL1A1 (collagentypeIalpha1).

### The diagnostic value of CALD1 was further evaluated using clinical samples from the GEO database

**Fig. 14:**
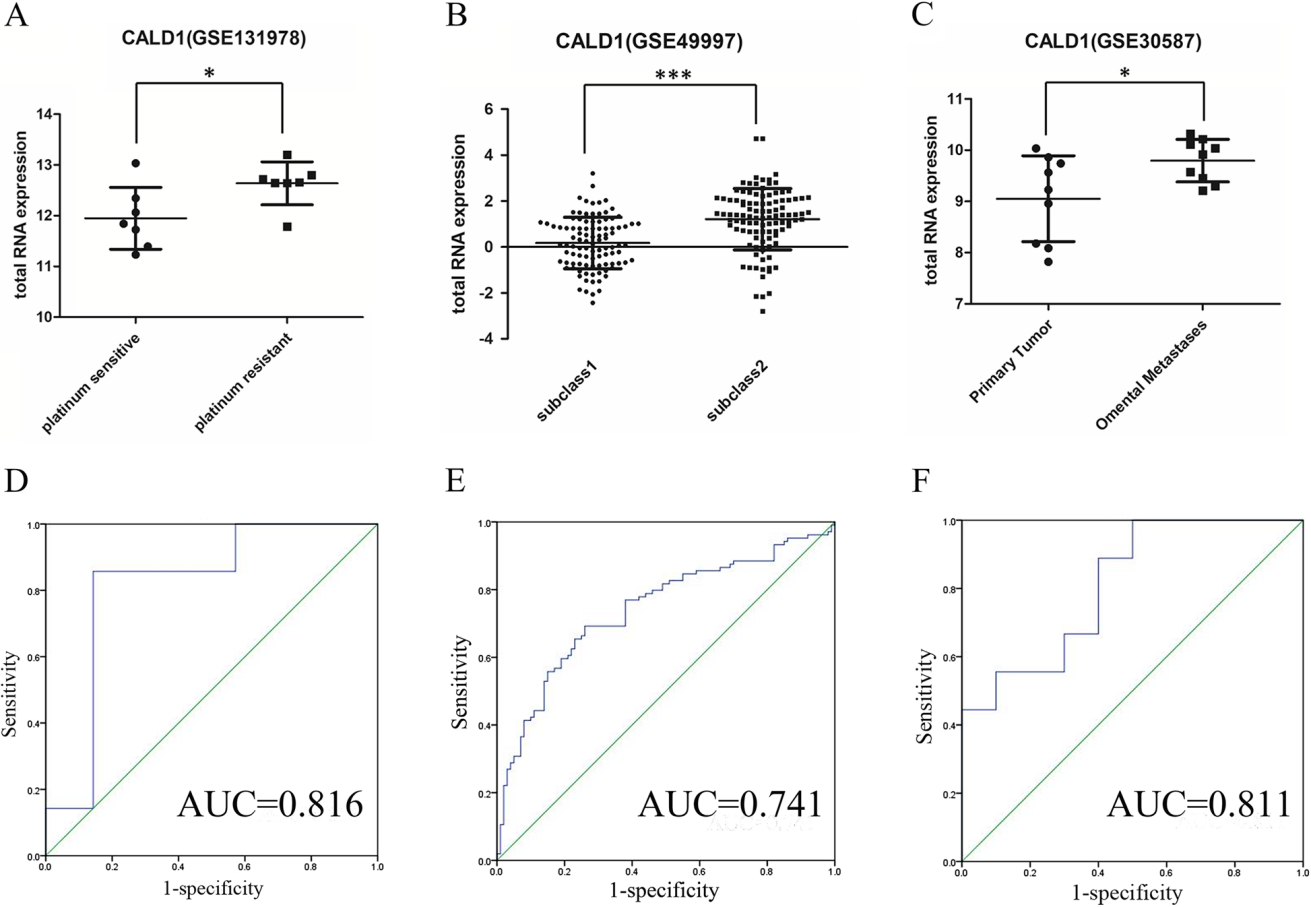
Total RNA expression of CALD1 were assessed in OC clinical specimens from datasets GSE131978 (**A**), GSE49997 (**B**) and GSE30587 (**C**). The corresponding AUC was 0.816, 0.741 and 0.811 respectively (DEF),which illustrated that CALD1 had diagnostic significance to distinguish platinum "resistant" and platinum "sensitive" patients, worse PFS patients, primary and omental metastases ovarian tumor. All data analyses were statistically significant. (* indicates *P* < 0.05, *** indicates *P* < 0.001)

To investigate whether the correlation between CALD1 and OC in clinical samples is consistent with previous cell-level findings, we retrieved three validated microarray data (GSE131978, GSE49997, GSE30587) from GEO and evaluated CALD1 expression. In GPL570 platform of GSE131978, by comparing RNA expression level between platinum "resistant" (PFI < 6 months) and platinum "sensitive" (PFI > 12 months) patients of high-grade serous ovarian carcinoma, CALD1 expression was up-regulated in platinum "resistant" and statistically significant (*P* = 0.03) (Fig. 14A). The RNA expression of CALD1 in GSE49997 showed that CALD1 expression was up-regulated in epithelial OC patients with worse PFS (HR 1.67, *P* = 0.005) and the outcomes demonstrated statistical significance (*P* < 0.0001) (Fig. 14B). The GSE30587 dataset was used to investigate the differential expression of CALD1 between matched ovarian primary tumors and omental metastases, which revealed a specific upregulation of CALD1 expression in omental metastatic ovarian tissue (*P* = 0.036) (Fig. 14C). Receiver Operating Characteristic (ROC) curve analysis of CALD1 was conducted using the SPSS 23.0 software. In GSE131978, we employed the datas of probe ID (212077_at) with the most significant *P*-value to construct ROC curve and area under the receiver operating characteristic curve (AUC) was calculated as 0.816(*P* < 0.05). Similarly, in GSE49997 and GSE30587 datasets, AUC values for CALD1 were determined as 0.741 and 0.811 respectively, all demonstrating statistical significance (*P* < 0.05) (Fig. 14DEF). Notably, all AUCs exceeded 70%, with some surpassing 80%. The results showed that expression levels of CALD1 could distinguish more aggressive OC well, those with good accuracy in diagnosing platinum resistant, worse PFS and omental metastases patients of OC.

### The up-regulation of CYR61 may contribute to the mechanism of platinum resistance in OC

**Fig. 15:**
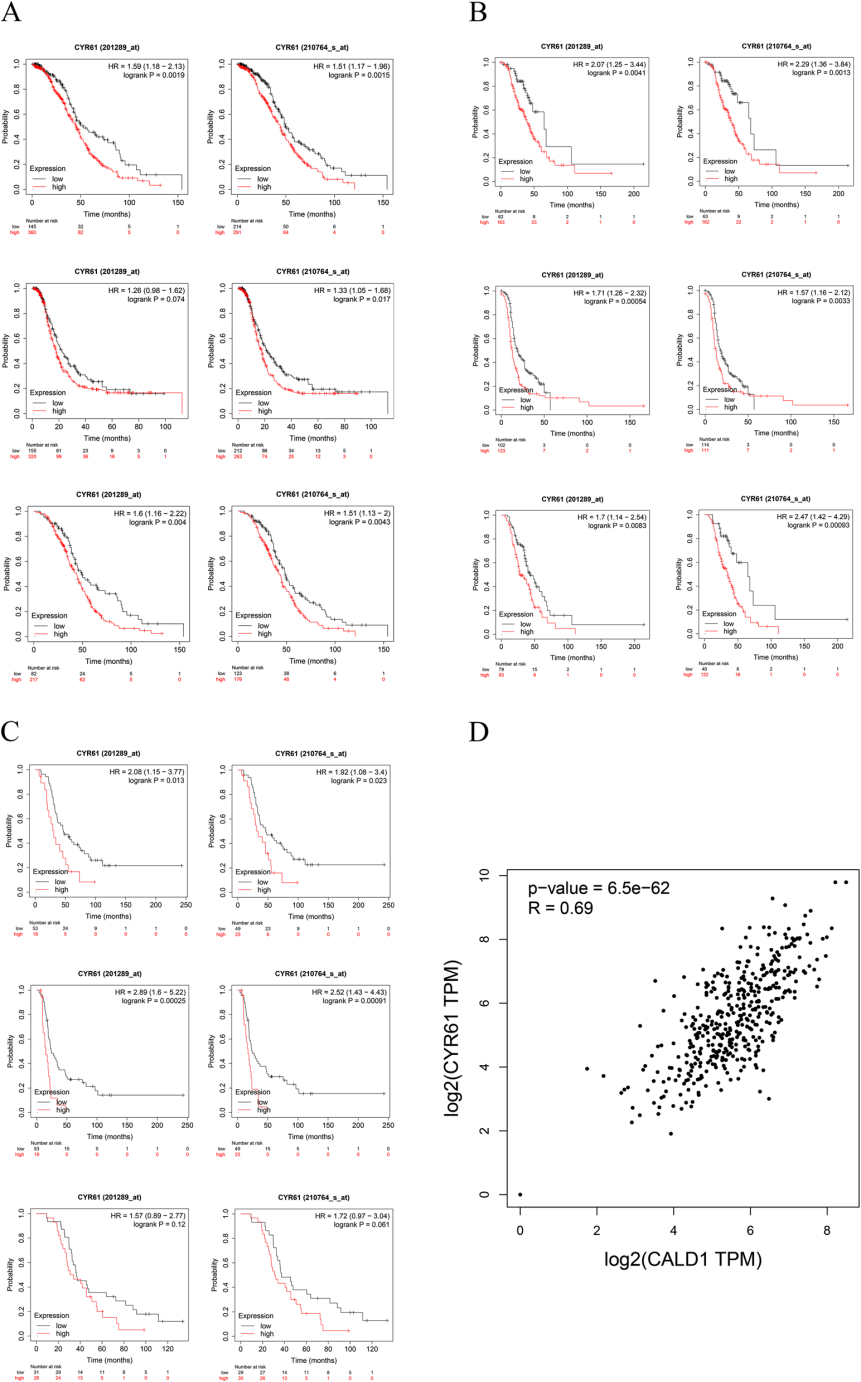
Correlation between the expression of CYR61 and OS, PFS, PPS of serous OC patients treated with platin-based chemotherapy in TCGA (**A**), GSE9891 (**B**) and GSE 26193 (**C**), which were displayed by 2 Affy ID (201289_at, 210764_s_at). Almost all of the *P*-values were less than 0.05. Using GEPIA 2, the positive correlation between CYR61 and CALD1 was further confirmed (**D**). The high degree of correlation between the two genes is striking (*P* = 6.5e-62, R = 0.69)

Among the 17 hub genes that directly interact with CALD1, CYR61 expression was significantly up-regulated in platinum-resistant OC cells, consistent with CALD1. This finding was supported by in vitro experimental data sets GSE45553 and GSE58470. Selecting the same conditions as CALD1, the prognostic values of CYR61 was assessed by Kaplan–Meier plotter. For patients receiving platinum chemotherapy, the high expression of CYR61 predicts poor OS, PFS, PPS using different probe sets in TCGA, GSE9891 and GSE26193 (Fig. 15 ABC). Most of P values of OS, PFS, PPS were statistically significant (*P* < 0.05). These findings suggest that, similar to CALD1, up-regulation of CYR61 may be indicative of poor prognosis and contribute to platinum-resistance mechanisms in OC. Analysis of patient data with ovarian serous adenocarcinoma from the TCGA database using GEPIA 2 confirmed a positive correlation between CYR61 and CALD1 (Fig. 15 D), supporting the potential association between CALD1 and CYR61 in platinum resistance of OC.

## Discussion

Because CALD1 is predominantly located in the fine myofilaments of smooth muscle, it was highly expressed in organs with abundant smooth muscle distribution, such as the colon, urinary bladder, gallbladder, testes, ovaries, endometrium, placenta and other smooth muscle tissues (Fig. 3). Due to its high expression in normal ovarian cells, CALD1 plays a crucial role in maintaining proper ovarian function. However, the expression of CALD1 in OC tissues does not exceed that of normal tissues. IHC analysis from the HPA database revealed that CALD1 was highly expressed in normal ovarian cells and exhibited strong or moderate expression levels across various types of ovarian malignancies including serous cystadenocarcinoma, mucinous cystadenocarcinoma and endometroid carcinoma of ovary (Fig. 6). It has been reported that CALD1 expression levels are reduced in epithelial OC, particularly in higher grade cases compared to benign ovarian tissue [23]. Analogously, CALD1 expression is elevated in normal tissue of other cancer types. In prostate cancer patients, the serum concentration of CALD1 was significantly lower than that of the general population [22]. Furthermore, glucocorticoid stimulation upregulates or overexpresses CALD1 as a regulatory protein involved in actomyosin contraction and actin filament stability within lung cancer cells. This promotes an increase in stress fibers and adhesive plaques while inhibiting cell migration [24].

However, it has been reported that, in carcinoma in situ, early stage of cancer, invasive cancer and cancer metastasis, the expression of CALD1 is higher than that in normal tissues and is associated with poor tumor prognosis [16, 21, 25–27]. Jiang Qifeng et al. [28] observed that the expression level of l-CaD was significantly lower or even absent in numerous non-metastatic cancer cells, whereas highly metastatic cancer cells with enhanced migration activity, such as HS578T and SNB-19, exhibited a substantial increase in l-CaD expression. Furthermore, their study demonstrated that phosphorylation of l-CaD serves as a crucial molecular switch regulating the heightened migration activity of metastatic cancer cells and modulating cytoskeletal changes. The robust expression and efficient phosphorylation cycle of l-CaD play pivotal roles in sustaining the heightened migratory activity of metastatic cancer cells. X Y Zhang group revealed, when OC cells developed peritoneal metastasis, fibroblasts had an altered protein expression pattern after being induced by OC cells, and participated in multiple cell processes resulting in tumor progression. CALD1 protein was significantly up-regulated [29]. In this study, TCGA data was used to analyze the relationship between the expression of CALD1 and the clinicopathological features of OC (Table 2). The results showed that clinical stage and venous invasion were significantly correlated with CALD1 expression. It was found that compared with the early stage of ovarian cancer, the expression of CALD1 increased significantly in stageIII-IV. The expression of this molecule was significantly higher in patients with vascular invasion than in patients without venous invasion. All these results support the conclusions of the literatures above, which is the high expression of CALD1 is associated with metastatic cancer cells. However, the precise role of CALD1 in various cancers remains a subject of controversy.

The CCLE database indicates that CALD1 expression is elevated in OC compared to most other tumors (Fig. 4). While more studies have identified CALD1 as a prognostic biomarker in bladder cancer [20, 30], gastric cancer [31], and colorectal cancer [21, 32], there is limited research on its role in OC, including its potential involvement in platinum-based chemotherapy resistance.

Up to now, platinum derived drugs are still the backbone of treating OC. In the course of most patients management, they treated with platinum-based chemotherapy develop drug resistance. Patients with platinum-resistant OC (PROC) have few therapeutic options. The treatment of platinum-resistant OC (PROC) is challenging [33]. Relevant studie shows off that, in a group of 1086 women affected by high-grade ovarian carcinoma treated with platinum-based chemotherapy in first-line, 81.2% of the patients progressed. Among women who experienced disease progression, majority of patients (61.3%) achieved a PFS of more than 6 months, while a minority (35.5%) N > 12 months [34, 35].

As the limitations of traditional treatment, it is particularly important to identify biomarkers associated with platinum resistance in OC and elucidate their role in the development of resistance. In this study, CALD1 was screened as a common molecule from 5 different datasets of experiments in vitro (Fig. 2). The up-regulation of CALD1 in platinum-resistant cells suggests that overexpression of this molecule may contribute to the development of chemotherapy resistance in OC cells.

In TCGA (Fig. 8), GSE9891 (Fig. 9) and GSE 26193 (Fig. 10), CALD1 was found that its high expression was related to poor prognosis of patients treated with platinum-based chemotherapy. Besides, in other datasets of diverse types of OC, CALD1 expression was up-regulated in the platinum "resistant" patients (Fig. 14A), the persons with poorer PFS (Fig. 14B), and the patients with omental metastases (Fig. 14C). Based on the AUC value of CALD1, it can be concluded that CALD1 showed excellent diagnosis rate as a potential diagnostic biomarker (Fig. 14 DEF).

Cancer stem cells (CSCs) are one of the few cell groups with infinite proliferation and self-renewal ability, which exist in most tumors and promote the occurrence and development, drug resistance, recurrence and metastasis of tumors after treatment, and are one of the mechanisms of the occurrence, development and poor prognosis of OC [36]. Traditional platinum-based combination chemotherapy can kill differentiated cells in the state of division and significantly reduce the volume of OC. However, OC stem cells are resistant to traditional primary treatment or have acquired resistance during treatment, and these residual tumor stem cells after initial chemotherapy become the root cause of OC recurrence and drug resistance. It has been reported that the effect of cisplatin in the treatment of OC is related to the changes of stem cell markers in residual tumors after chemotherapy, and the expression of CSCs markers is increased in chemotherapy resistant OC [37, 38]. The relationship between CALD1 expression and CSCs has not been reported. Further research is needed to determine if CALD1 can impact chemotherapy resistance in OC through the mechanism of CSCs.

**Fig. 16:**
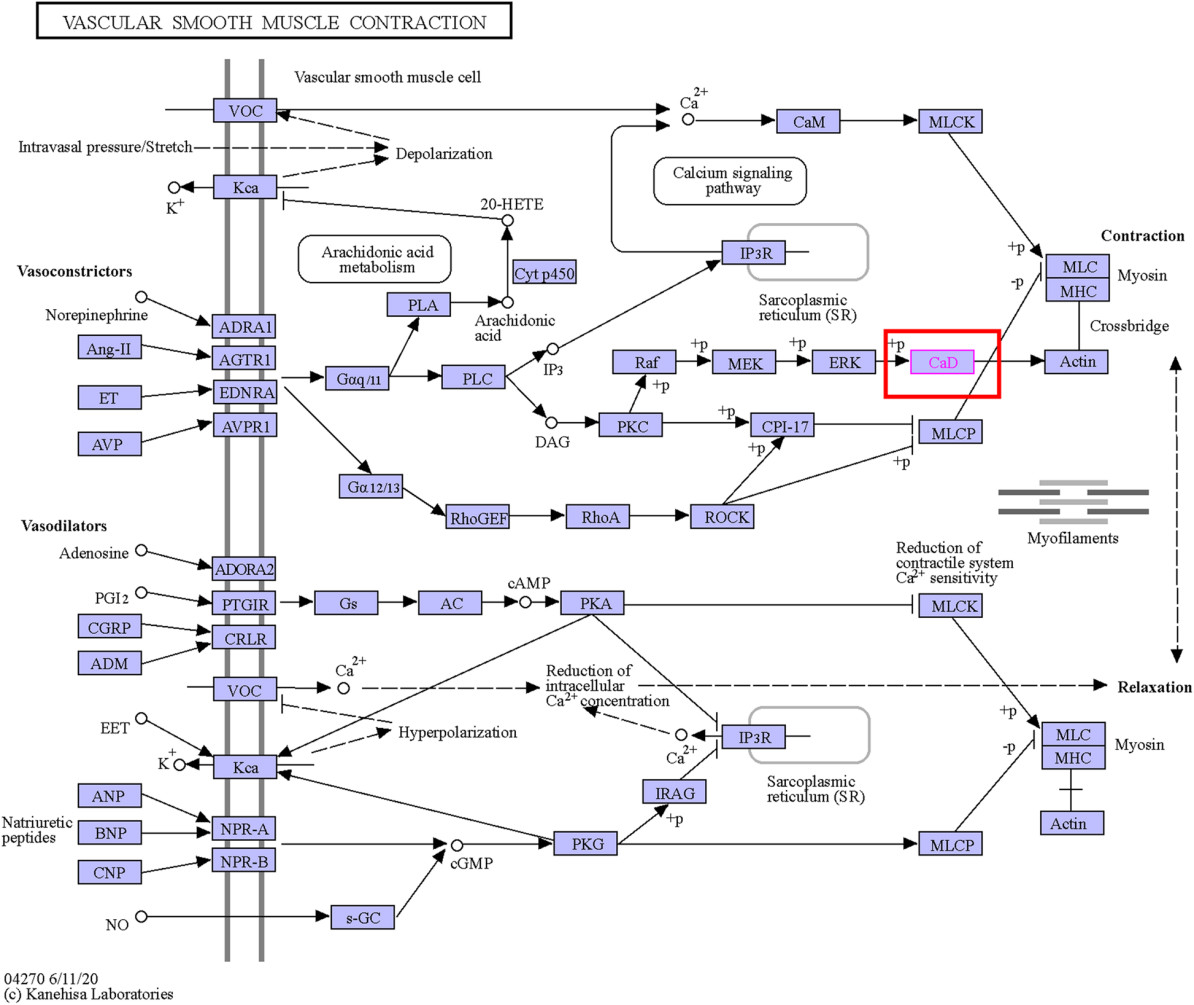
Signal pathways related to CALD1 mapped by KEGG database. CALD1 participates in vascular smooth muscle contraction and calcium signaling pathways. MAPK signaling pathway related proteins, such as MAPK and ERK, regulat CALD1 by phosphorylation, which regulates muscle contraction. The abbreviation CALD1 is CAD and marked in a red box

Through functional enrichment analysis in the LinkedOmics database, we found that, in gene sets of CALD1 and its co-expressed genes, majority of genes are enriched in the PI3K-Akt signaling pathway, displaying by enrichment categories of pathway_KEGG (Fig. 12). An increasing number of recent studies have demonstrated the association between the development of CSCs and the PI3K/Akt/mTOR signaling pathway. The available data currently suggest that targeting the PI3K/Akt/mTOR signaling pathway holds promise for the development of CSC-targeted drugs [39]. Dubrovska et al. [40] found that PTEN/PI3K/Akt pathway is critical for prostate cancer stem-like cell maintenance and that targeting PI3K signaling may be beneficial in prostate cancer treatment by eliminating prostate cancer stem-like cells. In both acute myeloid leukemia (AML) and acute lymphoblastic leukemia (ALL), activated PI3K upregulated ABCG2 expression and elevated percentage of cancer stem-like cells [41]. Currently, there is no literature to prove the correlation between the PI3K signaling pathway and CSCs in OC. Using the KEGG database (https://www.kegg.jp/), we found that CALD1 has no direct effect on the PI3K/Akt/mTOR signaling pathway. CALD1 mainly play a role in vascular smooth muscle contraction and calcium signaling pathways in map 04270 (Fig. 16) [42–44]. Meanwhile, MAPK signaling pathway related proteins, such as MAPK and ERK, regulat CALD1 by phosphorylation (Fig. 16). It has also been reported that CALD1 could participate in important physiological responses in tumors, which was related to ERK/MAPK signaling pathways [45, 46]. Increasing evidence indicates that NF-*κ*B, MAPK, PI3K, and EGF pathways are related to self-renewal. These intricate signaling pathways play a regulatory network role in the CSC niche. MAPK inhibitor have been used for both bulk hepatocellular carcinoma (HCC) and HCC CSC therapy, higher therapeutic efficacy were achieved [47]. Further research is needed to investigate whether CALD1 can affect the generation of CSCs and drug resistance in OC through the PI3K-Akt pathway or ERK/MAPK signaling pathways.

CYR61 (CNN1), a member of the cysteine rich 61/connective tissue growth factor/nephroblastoma over expressed (CYR61/CTFG/NOV) family of growth regulators (CNN), is a pro-angiogenic factor that mediates diverse roles in development, cell proliferation, and tumorigenesis [48]. Among genes that interact directly with CALD1, CYR61 was positively correlated with CALD1 (Fig. 13, Fig. 15). In platinum-resistant cells, CYR61 expression was up-regulated. Similarly to CALD1, high expression of CYR61 was associated with poor prognosis in OC patients treated with platinum-based chemotherapy in different databases (Fig. 15). Numerous studies have shown CYR61 might serve as a marker for different cancer, such as breast cancer [48], lung cancer [49], prostate cancer [50], etc. Our study further demonstrated that high expression of CALD1 was associated with poor prognosis of platinum-resistant OC, and revealed CALD1 could be used as a potential target for platinum-resistant OC. In the future, more investigations should be performed to confirm if CALD1 affects the survival of CSCs through the collation of PI3K signal or MAPK signal, and whether it interacts with the tumor-related molecule CYR61 to promote the progress of drug resistance. The precise molecular mechanisms of CALD1 for platinum-resistant need further studies to elucidate.

## Conclusion

In summary, we filtered through different databases to identify target molecule CALD1, which represents a potential therapeutic target for platinum-resistant OC. The altered expression of CALD1 was found to be associated with platinum resistance and poor prognosis in patients with platinum-treated OC. CALD1 has certain diagnostic value in different states of OC. It would enrich tumor marker members in the tumorigenesis of OC and provide more ideas for solving platinum resistance.

## Methods

### Gene Expression Omnibus (GEO) database

We searched the expression microarray datasets of platinum-based chemoresistance of OC from National Center for Biotechnology Information Gene Expression Omnibus database (GEO, http://www.ncbi.nlm.nih.gov/geo/). GEO is a public database providing functional genomic information from high-throughout gene expression, chips, and microarray data. Through searching in the GEO with key words “ovarian cancer”, “resistant” and “platinum”, we found 5 datasets about microarray gene expression of ovarian carcinoma platinum-sensitive and -resistant cell lines in vitro. The cell lines of enrolled datasets in our study are different OC cell lines, which include A2780, OVCAR-8, IGROV-1 etc. The 5 OC-related datasets were analyzed in the present study, namely GSE15372 [51] (expression data from cisplatin-sensitive A2780 and Round5 cisplatin-resistant A2780 cell lines),GSE33482 (expression data of 6 independent experiments from cisplatin-sensitive A2780 and cisplatin-resistant A2780 cell lines), GSE45553 [52] (expression data from cisplatin-sensitive OVCAR-8 and cisplatin-resistant OVCAR-8cell lines), GSE58470 [53] (gene expression of platinum-sensitive IGROV-1 and cisplatin-resistant IGROV-1 cell lines), GSE73935 [54] (expression data from wild type human OC W1 and cisplatin resistant human OC W1 cell lines. The human OC W1 cell line was established using OC tissue obtained from an untreated patient.). The GEO2R tool was employed to investigate differential gene expression. GEO2R, an interactive web-based application, facilitates the comparison of multiple sample groups within a GEO Series for the purpose of identifying genes that exhibit differential expression across experimental conditions [55].

### Venn plot

“*P* < 0.05 and Log (Fold Change) > 1 or Log (Fold Change) <  − 1” were defined as the thresholds for the screening of differential expression of genes in the enrolled 5 datasets. Compared with cisplatin-sensitive or parental cells lines, we obtained up-regulated and down-regulated DEGs of cisplatin-resistant cell lines across these five datasets. To identify co-expression genes, the up-regulated or down-regulated genes of five datasets were imported into the website Draw Venn Diagram (http://bioinformatics.psb.ugent.be/webtools/Venn/) respectively. The Venn plot was performed to collect the consensus genes from the five online databases.

### Analysis of The Human Protein Atlas (HPA) database

The HPA (https://www.proteinatlas.org/) is user-friendly online server, which contains the human transcriptomic and proteomic data in cells, tissues, and organs from human normal or pathological tissues via RNA sequencing (RNA-Seq) analysis and IHC [56]. In this study, through searching “CALD1” as term in HPA, the protein or RNA expression overview of CALD1 was analyzed in multiple tissues including ovary stroma cells. In addition, immunohistochemical results of CALD1 were searched in tissue-enhanced and PATHOLOGY for comparing the expression of CALD1 between normal ovarian stroma cells and ovarian malignant tumor.

### Analysis of The Cancer Cell Line Encyclopedia (CCLE) database

Using Cancer Cell Line Encyclopedia (CCLE) database, the RNA-Seq results were used to verify CALD1 expression in various cancer. CCLE database (https://portals.broadinstitute.org/ccle) is an effort to conduct comprehensive genetic characterization of a large panel of human cancer cell lines from individuals of various lineages and ethnicities [57].

### Pan cancer analysis of CALD1

The Pan cancer analysis of CALD1 was performed by GEPIA 2 (http://gepia2.cancer-pku.cn/#index) which is a tool for online bioinformatics analysis based on TCGA data. ANOVA was chosen as “Differential Methods” in Gene Expression Profile. The setting condition including, |Log2FC| Cutoff: 1, q-value Cutoff: 0.01. “Match TCGA normal and GTEx data” was chosen as Matched Normal data.

### Application of The University of ALabama at Birmingham CANcer data analysis Portal (UALCAN)

UALCAN (http://ualcan.path.uab.edu/analysis-prot.html) provides protein expression analysis option using data from Clinical Proteomic Tumor Analysis Consortium (CPTAC) and the International Cancer Proteogenome Consortium (ICPC) datasets. The protein expression for multiple cancers are available. The correlation between CALD1 expression and the stage of OC can be examined on this website, yielding results derived from TCGA dataset of ovarian serous cystadenocarcinoma.

### The Cancer Genome Atlas (TCGA) database analysis

We studied 379 OC patients with RNA-seq and clinical data from the TCGA (https://portal.gdc.cancer.gov) to analyze association between CALD1 expression and different clinicopathologic features. The main clinical characteristics of the OC patients are presented in Table 2, which include age at diagnosis (years), clinical stage, grade, longest dimension, anatomic neoplasm subdivision, residual tumor, lymphatic invasion, venous invasion. In the statistical analysis, a two-independent sample T-test was employed for bivariate comparisons, while a one-way ANOVA was utilized for examining multivariate relationships.

### Kaplan–Meier (KM) plotter analysis

The prognostic value of CALD1 mRNA expression was evaluated using an online database-Kaplan–Meier Plotter (www.kmplot.com), which contained gene expression datas and survival information of OC patients (Kaplan–Meier plotter [Ovarian] (kmplot.com)). To assess the OS, PFS, and PPS of OC patients treated with platinum-based chemotherapy, we selected "Serous" histology as the focus of this analysis due to its large sample size, and "Contains platin" as chemotherapy to restrict treatment group. Meanwhile, patient samples were split into two groups by median expression (high versus low expression) and assessed by Kaplan–Meier survival plots with hazard ratio (HR) (95% confidence intervals (CIs)) and log rank p value. All probe sets of CALD1 were selected for generating Kaplan–Meier plots, with the number-at-risk indicated below the main plots.

### Linked Omics database analysis

The LinkedOmics database (http://www.linkedomics.orglogin.php) [58] was utilized to screen for co-expression genes correlated with CALD1 expression. In this database, ovarian serous cystadenocarcinoma as the cancer type was chosen to analysis co-expression genes. The mRNA sequencing data from the TCGA database was employed, and Pearson's correlation coefficient was subsequently calculated for data analysis. Using LinkedOmics, the list of co-expression genes and volcano plots for analyzing of the co-expression genes associated with CALD1 could be showed. Additionally, functional enrichment analysis of CALD1 and its co-expression genes was performed using the LinkInterpreter module within LinkedOmics.

### Protein–Protein Interaction (PPI) analysis

A strong correlation between screened molecules is defined as an absolute value of Pearson correlation coefficient greater than 0.6. Co-expression genes of CALD1 were obtained from LinkedOmics database, and only strong correlation proteins (|r|≥ 0.6) were filtered for analysis using the String database to construct a PPI network [59]. In String database, we selected “Homo sapiens” from the available organism options. Software Cytoscape_v3.6.1 was used to identify proteins that interact directly with CALD1 and visualize the network [60].

### Analysis of clinical samples in GEO and construction of ROC curves for CALD1

To validate the consistency between the expression levels of target molecules identified from cell line datasets and clinical samples, we obtained clinical sample datasets of OC in various stages from GEO. The diagnostic and prognostic significance of CALD1 was further confirmed using these clinical samples. Data on CALD1 expression from GSE131978, GSE49997, and GSE30587 were downloaded from the profile graph of the GEO2R online program based on gene ID. Dot graphs were generated using GraphPad Prism with “mean with SD”. Additionally, an unpaired t-test in GraphPad Prism was employed to assess the significance between two groups in the validated GEO database. ROC and calculation of AUC for CALD1 was performed on GSE131978, GSE49997 and GSE30587 using SPSS 23.0 software (SPSS Statistics, IBM, Chicago, IL, USA). The AUCs > 70% was set as the cut-off value to determine the diagnostic significance of biomarker.

### Kaplan–Meier (KM) plotter analysis of co-expression gene CYR61 and correlation analysis of CYR61 and CALD1

Among the 17 hub genes, CYR61 was conformed that interact directly with CALD1. We observed significant up-regulation of CYR61 expression in platinum-resistant OC cells based on pre-existing datasets GSE45553 and GSE58470. Furthermore, the prognostic significance of CYR61 mRNA expression was assessed using Kaplan–Meier Plotter. Correlation between CYR61 and CALD1 was confirmed with GEPIA 2 database in TCGA. The spearman correlation coefficient was analyzed in Used Expression Datasets of “OV Tumer”.

### Statistical analysis

Statistical analyses and result visualization were performed using GEO2R online program, IBM SPSS Statistics 23, Graphpad Prism 5 and OmicShare tools (www.omicshare.com/tools). Kaplan–Meier Plotter (www.kmplot.com) were used to evaluate OS, PFS and PPS in OC patients treated with platinum-based drugs. A two-independent sample T-test was employed for bivariate comparisons, while a one-way ANOVA was utilized for examining multivariate relationships in correlation analysis. All P values were 2-tailed, and a *P* < 0.05 was considered statistically significant.

### Supplementary Information

**Additional file 1.** Co-expressed genes in conjunction with CALD1 analyzed by LinkedOmics database.

**Additional file 2.** 140 protein-coding genes strongly positive correlated with CALD1.

## Data Availability

The datasets generated and/or analysed during the current study are available in the GEO repository [https://www.ncbi.nlm.nih.gov/gds/?term=GSE15372; https://www.ncbi.nlm.nih.gov/gds/?term=GSE33482; https://www.ncbi.nlm.nih.gov/gds/?term=GSE45553; https://www.ncbi.nlm.nih.gov/gds/?term=GSE58470; https://www.ncbi.nlm.nih.gov/gds/?term=GSE73935; https://www.ncbi.nlm.nih.gov/gds/?term=GSE131978; https://www.ncbi.nlm.nih.gov/gds/?term=GSE49997; https://www.ncbi.nlm.nih.gov/gds/?term=GSE30587], TCGA database [https://portal.gdc.cancer.gov], CCLE database [https://depmap.org/portal/gene/CALD1?tab=overview], Kaplan–Meier Plotter [https://kmplot.com/analysis/index.php?p=service&cancer=ovar].

## Abbreviations

OC: Ovarian cancer
ROC: Receiver Operating Characteristic
AUC: Area under the receiver operating characteristic curve
GEO: Gene Expression Omnibus
HPA: Human Protein Atlas
CCLE: The Cancer Cell Line Encyclopedia
KM: Kaplan-Meier
OS: Overall survival
PFS: Progression-free survival
PPS: Post progression survival
HR: Hazard ratio
CIs: Confidence intervals
PPI: Protein-Protein Interaction
IHC: Immunohistochemistry
GSEA: Gene Set Enrichment Analysis
TCGA: The Cancer Genome Atlas
UALCAN: The University of ALabama at Birmingham CANcer data analysis Portal

## References

[1] Siegel RL, Miller KD, Jemal A (2019). Cancer statistics, 2019. CA Cancer J Clin.

[2] Siegel RL, Miller KD, Jemal A (2018). Cancer statistics. CA Cancer J Clin..

[3] Henderson JT, Webber EM, Sawaya GF (2018). Screening for ovarian cancer: updated evidence report and systematic review for the US preventive services task force. JAMA.

[4] Bogani G, Matteucci L, Tamberi S, Arcangeli V, Ditto A, Maltese G (2017). The impact of number of cycles of neoadjuvant chemotherapy on survival of patients undergoing interval debulking surgery for stage IIIC-IV unresectable ovarian cancer: results from a multi-institutional study. Int J Gynecol Cancer.

[5] Friedlander ML, Stockler MR, Butow P, King MT, McAlpine J, Tinker A (2013). Clinical trials of palliative chemotherapy in platinum-resistant or -refractory ovarian cancer: time to think differently?. J Clin Oncol.

[6] Bogani G, Lopez S, Mantiero M, Ducceschi M, Bosio S, Ruisi S (2020). Immunotherapy for platinum-resistant ovarian cancer. Gynecol Oncol.

[7] Rahma OE, Duffy A, Liewehr DJ, Steinberg SM, Greten TF (2013). Second-line treatment in advanced pancreatic cancer: a comprehensive analysis of published clinical trials. Ann Oncol.

[8] Wagner AD, Syn NL, Moehler M, Grothe W, Yong WP, Tai BC (2017). Chemotherapy for advanced gastric cancer. Cochr Database Syst Rev..

[9] Mason SR, Willson ML, Egger SJ, Beith J, Dear RF, Goodwin A (2023). Platinum-based chemotherapy for early triple-negative breast cancer. Cochr Database Syst Rev..

[10] Scatchard K, Forrest JL, Flubacher M, Cornes P, Williams C (2012). Chemotherapy for metastatic and recurrent cervical cancer. Cochr Database Syst Rev..

[11] Chandra A, Pius C, Nabeel M, Nair M, Vishwanatha JK, Ahmad S (2019). Ovarian cancer: current status and strategies for improving therapeutic outcomes. Cancer Med.

[12] Yang L, Xie HJ, Li YY, Wang X, Liu XX, Mai J (2022). Molecular mechanisms of platinum-based chemotherapy resistance in ovarian cancer (Review). Oncol Rep.

[13] Yilmaz M, Christofori G (2009). EMT, the cytoskeleton, and cancer cell invasion. Cancer Metastasis Rev.

[14] Ni S, Hu J, Duan Y, Shi S, Li R, Wu H (2013). Down expression of LRP1B promotes cell migration via RhoA/Cdc42 pathway and actin cytoskeleton remodeling in renal cell cancer. Cancer Sci.

[15] Jiang P, Enomoto A, Takahashi M (2009). Cell biology of the movement of breast cancer cells: intracellular signalling and the actin cytoskeleton. Cancer Lett.

[16] Chang KP, Wang CL, Kao HK, Liang Y, Liu SC, Huang LL (2013). Overexpression of caldesmon is associated with lymph node metastasis and poorer prognosis in patients with oral cavity squamous cell carcinoma. Cancer.

[17] Zheng PP, van der Weiden M, Kros JM (2007). Hela l-CaD is implicated in the migration of endothelial cells/endothelial progenitor cells in human neoplasms. Cell Adh Migr.

[18] Hodgson A, Swanson D, Tang S, Dickson BC, Turashvili G. Gene fusions characterize a subset of uterine cellular leiomyomas. Genes Chromosomes Cancer. 2020:1–9. 10.1002/gcc.22888 32677742

[19] Hamza A, Guo CC (2020). Perivascular epithelioid cell tumor of the urinary bladder: a systematic review. Int J Surg Pathol.

[20] Liu Y, Wu X, Wang G, Hu S, Zhang Y, Zhao S (2019). CALD1, CNN1, and TAGLN identified as potential prognostic molecular markers of bladder cancer by bioinformatics analysis. Medicine (Baltimore).

[21] Zhao B, Baloch Z, Ma Y, Wan Z, Huo Y, Li F (2019). Identification of potential key genes and pathways in early-onset colorectal cancer through bioinformatics analysis. Cancer Control.

[22] Xu L, Lee JR, Hao S, Ling XB, Brooks JD, Wang SX (2019). Improved detection of prostate cancer using a magneto-nanosensor assay for serum circulating autoantibodies. PLoS ONE.

[23] Boljevic I, Malisic E, Milovic Kovacevic M, Jovanic I, Jankovic R (2020). Expression levels of genes involved in cell adhesion and motility correlate with poor clinicopathological features of epithelial ovarian cancer. J buon.

[24] Mayanagi T, Morita T, Hayashi K, Fukumoto K, Sobue K (2008). Glucocorticoid receptor-mediated expression of caldesmon regulates cell migration via the reorganization of the actin cytoskeleton. J Biol Chem.

[25] Kim KH, Yeo SG, Kim WK, Kim DY, Yeo HY, Hong JP (2012). Up-regulated expression of l-caldesmon associated with malignancy of colorectal cancer. BMC Cancer.

[26] Yokota M, Kojima M, Higuchi Y, Nishizawa Y, Kobayashi A, Ito M (2016). Gene expression profile in the activation of subperitoneal fibroblasts reflects prognosis of patients with colon cancer. Int J Cancer.

[27] Lee MS, Lee J, Kim JH, Kim WT, Kim WJ, Ahn H (2015). Overexpression of caldesmon is associated with tumor progression in patients with primary non-muscle-invasive bladder cancer. Oncotarget.

[28] Jiang QF, Cai SX, Yan XQ (2010). The effect of caldesmon phosphorylation on metastatic tumor cell mobility. Prog Biochem Biophys.

[29] Zhang XY, Hong SS, Zhang M, Cai QQ, Zhang MX, Xu CJ (2018). Proteomic alterations of fibroblasts induced by ovarian cancer cells reveal potential cancer targets. Neoplasma.

[30] Du Y, Jiang X, Wang B, Cao J, Wang Y, Yu J (2021). The cancer-associated fibroblasts related gene CALD1 is a prognostic biomarker and correlated with immune infiltration in bladder cancer. Cancer Cell Int.

[31] Liu Y, Xie S, Zhu K, Guan X, Guo L, Lu R (2021). CALD1 is a prognostic biomarker and correlated with immune infiltrates in gastric cancers. Heliyon.

[32] Zheng H, Bai Y, Wang J, Chen S, Zhang J, Zhu J (2021). Weighted gene co-expression network analysis identifies CALD1 as a biomarker related to M2 macrophages infiltration in stage III and IV mismatch repair-proficient colorectal carcinoma. Front Mol Biosci.

[33] El Bairi K, Al Jarroudi O, Afqir S (2021). Revisiting antibody-drug conjugates and their predictive biomarkers in platinum-resistant ovarian cancer. Semin Cancer Biol.

[34] Lindemann K, Gao B, Mapagu C, Fereday S, Emmanuel C, Alsop K (2018). Response rates to second-line platinum-based therapy in ovarian cancer patients challenge the clinical definition of platinum resistance. Gynecol Oncol.

[35] Lindemann K, Christensen RD, Vergote I, Stuart G, Izquierdo MA, Kærn J (2012). First-line treatment of advanced ovarian cancer with paclitaxel/carboplatin with or without epirubicin (TEC versus TC)–a gynecologic cancer intergroup study of the NSGO, EORTC GCG and NCIC CTG. Ann Oncol.

[36] Najafi M, Farhood B, Mortezaee K (2019). Cancer stem cells (CSCs) in cancer progression and therapy. J Cell Physiol.

[37] Latifi A, Abubaker K, Castrechini N, Ward AC, Liongue C, Dobill F (2011). Cisplatin treatment of primary and metastatic epithelial ovarian carcinomas generates residual cells with mesenchymal stem cell-like profile. J Cell Biochem.

[38] Steg AD, Bevis KS, Katre AA, Ziebarth A, Dobbin ZC, Alvarez RD (2012). Stem cell pathways contribute to clinical chemoresistance in ovarian cancer. Clin Cancer Res.

[39] Xia P, Xu XY (2015). PI3K/Akt/mTOR signaling pathway in cancer stem cells: from basic research to clinical application. Am J Cancer Res.

[40] Dubrovska A, Kim S, Salamone RJ, Walker JR, Maira SM, García-Echeverría C (2009). The role of PTEN/Akt/PI3K signaling in the maintenance and viability of prostate cancer stem-like cell populations. Proc Natl Acad Sci U S A.

[41] Huang FF, Wu DS, Zhang L, Yu YH, Yuan XY, Li WJ (2013). Inactivation of PTEN increases ABCG2 expression and the side population through the PI3K/Akt pathway in adult acute leukemia. Cancer Lett.

[42] Kanehisa M, Goto S (2000). KEGG: Kyoto encyclopedia of genes and genomes. Nucleic Acids Res.

[43] Kanehisa M (2019). Toward understanding the origin and evolution of cellular organisms. Protein Sci.

[44] Kanehisa M, Furumichi M, Sato Y, Kawashima M, Ishiguro-Watanabe M (2023). KEGG for taxonomy-based analysis of pathways and genomes. Nucleic Acids Res.

[45] Ilyas U, Zaman SU, Altaf R, Nadeem H, Muhammad SA (2020). Genome wide meta-analysis of cDNA datasets reveals new target gene signatures of colorectal cancer based on systems biology approach. J Biol Res (Thessalon).

[46] Qi F, Wang Y, Yu B, Li F (2023). Identification of RECK as a protective prognostic indicator and a tumor suppressor through regulation of the ERK/MAPK signaling pathway in gastric cancer. J Transl Med.

[47] Duan H, Liu Y, Gao Z, Huang W (2021). Recent advances in drug delivery systems for targeting cancer stem cells. Acta Pharm Sin B.

[48] Menéndez JA, Mehmi I, Griggs DW, Lupu R (2003). The angiogenic factor CYR61 in breast cancer: molecular pathology and therapeutic perspectives. Endocr Relat Cancer.

[49] Zhu Y, Almuntashiri S, Han Y, Wang X, Somanath PR, Zhang D (2020). The roles of CCN1/CYR61 in pulmonary diseases. Int J Mol Sci.

[50] Terada N, Kulkarni P, Getzenberg RH (2012). Cyr61 is a potential prognostic marker for prostate cancer. Asian J Androl.

[51] Li M, Balch C, Montgomery JS, Jeong M, Chung JH, Yan P (2009). Integrated analysis of DNA methylation and gene expression reveals specific signaling pathways associated with platinum resistance in ovarian cancer. BMC Med Genomics.

[52] Chowanadisai W, Messerli SM, Miller DH, Medina JE, Hamilton JW, Messerli MA (2016). Cisplatin resistant spheroids model clinically relevant survival mechanisms in ovarian tumors. PLoS ONE.

[53] Arrighetti N, Cossa G, De Cecco L, Stucchi S, Carenini N, Corna E (2016). PKC-alpha modulation by miR-483-3p in platinum-resistant ovarian carcinoma cells. Toxicol Appl Pharmacol.

[54] Januchowski R, Zawierucha P, Ruciński M, Zabel M (2014). Microarray-based detection and expression analysis of extracellular matrix proteins in drug-resistant ovarian cancer cell lines. Oncol Rep.

[55] Davis S, Meltzer PS (2007). GEOquery: a bridge between the Gene Expression Omnibus (GEO) and BioConductor. Bioinformatics.

[56] Thul PJ, Lindskog C (2018). The human protein atlas: a spatial map of the human proteome. Protein Sci.

[57] Barretina J, Caponigro G, Stransky N, Venkatesan K, Margolin AA, Kim S (2012). The cancer cell line encyclopedia enables predictive modelling of anticancer drug sensitivity. Nature.

[58] Vasaikar SV, Straub P, Wang J, Zhang B (2018). LinkedOmics: analyzing multi-omics data within and across 32 cancer types. Nucleic Acids Res.

[59] Szklarczyk D, Franceschini A, Wyder S, Forslund K, Heller D, Huerta-Cepas J (2015). STRING v10: protein-protein interaction networks, integrated over the tree of life. Nucleic Acids Res..

[60] Shannon P, Markiel A, Ozier O, Baliga NS, Wang JT, Ramage D (2003). Cytoscape: a software environment for integrated models of biomolecular interaction networks. Genome Res.

